# Insights into the microbial life in silica-rich subterranean environments: microbial communities and ecological interactions in an orthoquartzite cave (Imawarì Yeuta, Auyan Tepui, Venezuela)

**DOI:** 10.3389/fmicb.2022.930302

**Published:** 2022-09-23

**Authors:** Daniele Ghezzi, Lisa Foschi, Andrea Firrincieli, Pei-Ying Hong, Freddy Vergara, Jo De Waele, Francesco Sauro, Martina Cappelletti

**Affiliations:** ^1^Department of Pharmacy and Biotechnology, University of Bologna, Bologna, Italy; ^2^Laboratory of NanoBiotechnology, IRCCS Istituto Ortopedico Rizzoli, Bologna, Italy; ^3^Division of Biological and Environmental Science and Engineering, King Abdullah University of Science and Technology (KAUST), Thuwal, Saudi Arabia; ^4^Teraphosa Exploring Team, Puerto Ordaz, Venezuela; ^5^La Venta Geographic Explorations Association, Treviso, Italy; ^6^Department of Biological Geological and Environmental Sciences, University of Bologna, Bologna, Italy

**Keywords:** cave microbiology, *Actinobacteriota*, *Acidobacteriota*, oligotrophic environment, silica speleothems, co-occurrence network, subterranean microbiome

## Abstract

Microbial communities inhabiting caves in quartz-rich rocks are still underexplored, despite their possible role in the silica cycle. The world’s longest orthoquartzite cave, Imawarì Yeuta, represents a perfect arena for the investigation of the interactions between microorganisms and silica in non-thermal environments due to the presence of extraordinary amounts of amorphous silica speleothems of different kinds. In this work, the microbial diversity of Imawarì Yeuta was dissected by analyzing nineteen samples collected from different locations representative of different silica amorphization phases and types of samples. Specifically, we investigated the major ecological patterns in cave biodiversity, specific taxa enrichment, and the main ecological clusters through co-occurrence network analysis. Water content greatly contributed to the microbial communities’ composition and structures in the cave leading to the sample clustering into three groups DRY, WET, and WATER. Each of these groups was enriched in members of *Actinobacteriota*, *Acidobacteriota*, and *Gammaproteobacteria*, respectively. Alpha diversity analysis showed the highest value of diversity and richness for the WET samples, while the DRY group had the lowest. This was accompanied by the presence of correlation patterns including either orders belonging to various phyla from WET samples or orders belonging to the *Actinobacteriota* and *Firmicutes* phyla from DRY group samples. The phylogenetic analysis of the dominant species in WET and DRY samples showed that *Acidobacteriota* and *Actinobacteriota* strains were affiliated with uncultured bacteria retrieved from various oligotrophic and silica/quartz-rich environments, not only associated with subterranean sites. Our results suggest that the water content greatly contributes to shaping the microbial diversity within a subterranean quartzite environment. Further, the phylogenetic affiliation between Imawarì Yeuta dominant microbes and reference strains retrieved from both surface and subsurface silica- and/or CO_2_/CO-rich environments, underlines the selective pressure applied by quartz as rock substrate. Oligotrophy probably in association with the geochemistry of silica/quartz low pH buffering activity and alternative energy sources led to the colonization of specific silica-associated microorganisms. This study provides clues for a better comprehension of the poorly known microbial life in subsurface and surface quartz-dominated environments.

## Introduction

Bacteria and archaea are numerically the most dominant and ubiquitous organisms of the Earth’s surface and subsurface (Merino et al., 2019). They can thrive in a wide variety of environmental conditions, overpowering barriers that are not compatible with life of higher organisms, including low and high temperatures, pH, radiation, pressure, salinity, absence of light, and nutrient limitation. In this context, dark and oligotrophic caves host abundant and complex microbial communities, whose structures are regulated by mechanisms that are distinct from those at the Earth’s surface. The subsurface is estimated to house 50%–87% of the Earth’s microorganisms (Kallmeyer et al., 2012; Magnabosco et al., 2018), most of which are still unknown or understudied. This aspect makes caves unique ecosystems suitable to study the evolution of microbial life and the survival mechanisms in the absence of primary productivity associated with sunlight and photosynthesis (Barton and Northup, 2007; Northup et al., 2011).

In caves, microbes take advantage of the interaction with the rock substrate to acquire essential elements for growth. Despite the nutrient-limited conditions, caves contain surprisingly diverse microbial communities with compositions that are influenced by a series of geochemical parameters including the host rock composition and other environmental factors such as temperature, organic carbon availability, and humidity (Barton et al., 2007). Nevertheless, cave microbiome studies have focused on limited amounts of samples that rarely allow detailed ecological correlation analyses. Furthermore, most of these studies focused on microbial diversity in carbonate caves (Brannen-Donnelly and Engel, 2015; Wu et al., 2015; Alonso et al., 2018; D’Angeli et al., 2019; Paun et al., 2019; Zhu et al., 2019). On the other hand, very little is known about the environmental variables influencing the microbial community composition and therefore the interactions between microbes and silica in quartz-dominated caves carved in orthoquartzites or metaquartzites (Barton et al., 2014; Sauro et al., 2018).

The importance of studying microbial interactions with silica resides in the fact that silicon is the seventh most abundant element in the universe and the second most abundant element on Earth, after oxygen. It can be found in the form of silicates, aluminosilicates, and crystalline and amorphous silicon dioxide (e.g., quartz and amorphous silica, respectively). The knowledge of the processes involved in the silica amorphization (transformation of crystalline silica into amorphous silica), dissolution, and precipitation is of great interest for the comprehension of the formation of ancient natural quartz-rich environments (Riquelme et al., 2015; Wray and Sauro, 2017; Sauro et al., 2018; Miller et al., 2022). Microbes are known to be involved in silica mineral dissolution and precipitation although in non-hydrothermal environments, the molecular and biochemical mechanisms are still unclear (Miller et al., 2014; Sauro et al., 2018). These processes leave traces of microbial features or metabolic activity in the rock record that are considered biosignatures valuable for astrobiology as potential analogs of silica-rich rocks detected on Mars (Cady and Farmer, 1996; Rice et al., 2010; Northup et al., 2011; Ruff and Farmer, 2016).

Quartzite caves in Venezuelan tepuis (i.e., orthoquartzite table mountains), represent an excellent natural laboratory to investigate microbe-mineral interactions in dark, low-temperature Si-rich environments. In particular, Imawarì Yeuta cave is composed of 98% of silica in the form of α-quartz and minor amounts of amorphous silica, i.e., opal-A, opal-G (Sauro et al., 2018; Ghezzi et al., 2021b). The cave was discovered in the Auyan Tepui in 2013 and is considered one of the most ancient caves in the world (Sauro et al., 2013). It hosts an extraordinary amount and variety of amorphous silica speleothems whose origin seems to be associated with biological activity. In fact, the absence of extreme chemical (pH) and/or physical (temperature, pressure) variations that would allow abiotic speleothem formation suggests possible biological mediation (Aubrecht et al., 2012; Sauro et al., 2018). Thus, the interaction between microbes and the orthoquartzite rocky substrates seems to contribute to this impressive speleothem formation process. In this regard, our previous works described the shifting of the composition/structure of microbial communities associated with some speleothems characterized by distinct silica amorphization phases (Sauro et al., 2018; Ghezzi et al., 2021b). Imawarì Yeuta provides a unique window to further investigate the microbial ecology of quartz/silica-rich environments under aphotic and non-thermal conditions. In addition to the absence of light, the isolation of the deepest Imawarì Yeuta cave zones from the exterior atmosphere determines a general low nutrient availability and low organic carbon sources, associable to highly selective oligotrophic conditions (Mecchia et al., 2014; Sauro et al., 2018).

In this work, the microbial diversity in Imawarì Yeuta cave is described by performing Illumina sequencing of 16S rRNA hypervariable regions on several samples collected from different cave niches. Statistical and correlation analyses were performed to investigate the environmental parameters driving the microbial community structure and composition in this silica-dominated cave. Phylogenetic analyses of computationally reconstructed near-full-length 16S rRNA genes further provided deeper inferences to the affiliation of the poorly classified microbes that dominate representative niches within the cave.

## Materials and methods

### Sample collection

**Figure 1:**
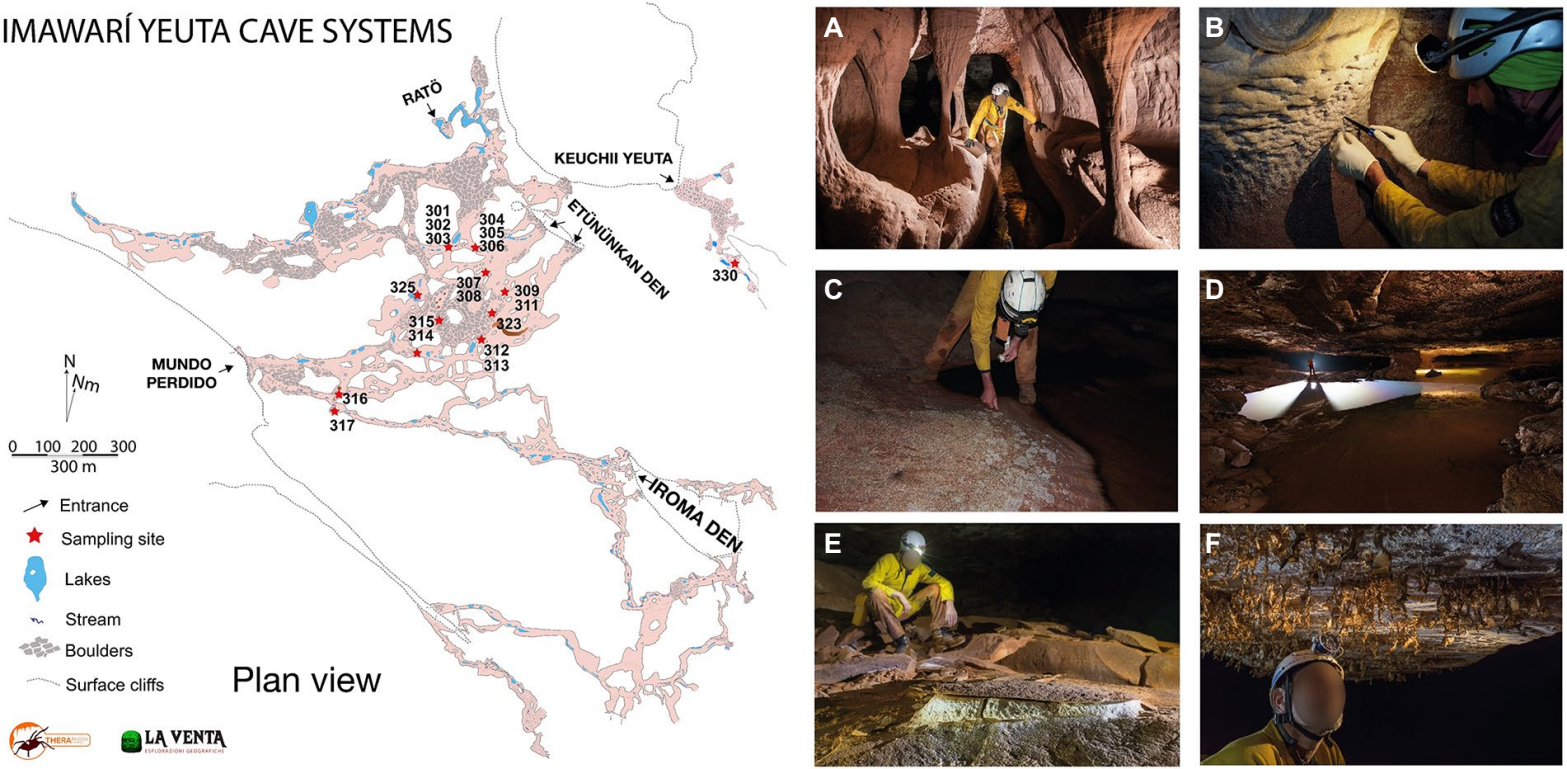
Map of Imawarì Yeuta cave and collection sites and examples of different geochemical, morphological, and hydrological environments in the cave. **(A)** Orthoquartzite columns and eroded walls are typical of hydrologically active branches of the cave, as those where sample Ay317 has been collected (photo La Venta-Alessio Romeo). **(B)** Cave walls undergoing quartz amorphization are usually covered by a wet paste of Opal-A, as in the sample location of Ay301 and Ay302 (photo La Venta-Francesco Lo Mastro). **(C)** Polished cave floors on a side of a stream are covered by whitish patches of biological material, as for Ay305 (photo La Venta-Francesco Lo Mastro). **(D)** Ponds of water covered by iridescent patinas and floating biological material, as for samples Ay303 and Ay314 (photo La Venta-Robbie Shone). **(E)** Deposits of powdery dry sulfates on the cave floors as for Ay308 and Ay315 (photo La Venta-Riccardo de Luca). **(F)** Fragile molds of silica resembling spider webs hanging from cave roofs, as for sample Ay323 (photo La Venta-Riccardo de Luca).

Samples have been collected in Imawarì Yeuta cave system during two field campaigns in March 2014 and March 2016. The cave system opens with seven different entrances at about 1,830 m a.s.l. in the northern-east sector of the Auyan Tepui massif (Sauro et al., 2013). Previous studies have shown that the cave is carved within Precambrian orthoquartzites of the Mataui Formation (Sauro, 2014) with an estimated speleogenetic age of over 30–40 million years (Mecchia et al., 2019). Cave conduits have been mapped for 23,079 m of length with 7 entrances, with an average depth of 80–100 m from the surface. Nineteen samples were collected from biofilms on water ponds, quartzite host rocks, sediments, and speleothems visible on the pavement, walls, and ceiling in different sites within the cave (Figure 1; Supplementary Table S1). The sampling strategy aimed to collect the most representative samples of different geochemical environments (niches) along the same cave system. However, each sample was kept to a minimum volume equivalent of 0.8 ml to a maximum of 30 ml in order to limit the damage to the very delicate cave deposits and environments. After scraping/collection with sterilized tools, all samples were stored in Eppendorf tubes and those designed for the molecular analysis were filled with LifeGuard RNA solution. The transport from the site to the lab was carried out in a portable fridge, then samples were stored at −80°C until analysis which happened within a few months after the expeditions.

### Geochemical and environmental analyses

Given the different nature of the samples (waters, sediments, mineral aggregates, rocks), the overall environmental and geochemical characterization was performed using different approaches. The water content of all samples was determined by measuring the water loss after drying them at 80°C to a constant weight. Temperature (T) and acidity (pH) of water samples were measured by handheld field instruments (HI991301 from Hanna Instruments, Italy) after calibration on site. Accuracy was 0.1°C and 0.01, respectively. In WATER and WET samples, pH was measured on site also with pH stripes with the range 2–9 and 0.5 pH unit increments (Macherey Nagel 92118), while T was measured through a portable probe thermometer (Hanna Instruments, Italy). For sediments, rocks, and mineral aggregates, pH was measured by resuspending the samples in distilled water and using pH stripes as previously described (Barton et al., 2014). Dissolved silica concentration (DSi) in waters was measured by using a field colorimetric test kit (Aquaquant 14410 Silicon – Merck) that allows the determination of silica in the concentration range 0.01–0.25 mg L^−1^ with an error <20%. The water samples presenting concentrations higher than 0.25 mg L^−1^ were diluted with distilled water and then analyzed. Results were expressed following the convention of representing dissolved silica as the oxide SiO_2_. To determine dissolved elements through inductively coupled plasma-mass spectrometry (ICP-MS) analyses in the laboratory, double water samples were collected in streams and ponds at Imawarì Yeuta in March 2013: a 250 ml bottle of untreated and unfiltered water, and a 100 ml bottle of 0.45 micron-filtered and 1 ml 65% HNO_3_ acid-preserved water. ICP-MS was applied (method EPA 6020 A) for determination of multi-elemental sub μg L^−1^ concentrations (Al, Sb, As, Ba, Cd, Ca, Fe, Mg, Pb, K, Na, Zn) for which the recovery of the Laboratory Control Sample (LCS) resulted between 85% and 115%, as expected by the method lines. Anion Chromatography (method EPA 9056 A) was used to determine chloride, fluoride, nitrate, and sulfate in the solution. NH_4_^+^ concentration was measured on the untreated sample with the method APAT CNR IRSA 4030 A2 MAN 292003. Analyses were carried out as described in Mecchia et al. (2014).

X-Ray Fluorescence (XRF) analysis was carried out for samples that had enough solid material (15 g) to conduct the analysis (7 samples out of 19). Bulk chemical analyses were conducted by a wave dispersive X-ray fluorescence spectrometer (WD-XRF) operating at the BIGEA department, University of Bologna (Italy). Ultra-fine powdered samples were mounted on rounded boric acid casts (~5 cm diameter, ~0.5 cm height), which were prepared according to the matrix correction method (Sauro et al., 2018). Thirty-five international reference materials were used for calibrating the raw results, allowing an accuracy better than 5% for elements >10 ppm, and between 10% and 15% for elements <10 ppm. Bulk XRD analysis was performed on all sediments, rocks, and mineral aggregate samples to identify the major mineral phase. Mineral phases were investigated by a Philips PW3710 X-Ray diffractometer (current: 20 mA, voltage: 40 kV, range 2*θ*: 5°–80°, step size: 0.02° 2*θ*, time per step: 2 s) as described by De Waele et al. (2017). Acquisition and processing of data was carried out using the Philips High Score software package. Major minerals are indicated in Supplementary Table S1, whereas for samples missing XRF analysis, prevalent chemical elements have been indicated based on mineral formulas. EDS analysis has been further used to confirm the prevalent composition of each sample as described by Sauro et al. (2018).

### DNA extraction, V4-V5 16S rRNA gene amplification, and sequencing

Nineteen cave samples were extracted for their total DNA using the PowerSoil DNA Isolation Kit (Qiagen) with slight modifications implementing pretreatments with proteinase K and lysozyme at 37°C, followed by an additional lysis step using a solution of sodium dodecyl sulfate (SDS; Cappelletti et al., 2016). To provide amplicon for Illumina MiSeq analysis, the total DNA was amplified for the V4-V5 hypervariable region of 16S rRNA gene with universal forward 515F (5′-Illumina overhang-GTGYCAGCMGCCGCGGTA-3′) and reverse 907R (5′-Illumina overhang-CCGTCAATTCMTTTRAGTTT-3′) primers (IDT DNA Technologies). The PCR reaction mixture contained 10 ng of total DNA, 1x Takara Ex Taq buffer with MgCl_2_ (10x, Takara Bio Inc., Tokyo, Japan), dNTP mix 200 μM, primers 500 nM, and Takara Ex Taq Polymerase 0.5 U and water (Lichrosolv^®^; Merck, Darmstadt, Germany) up to a total volume of 50 μl (Ghezzi et al., 2021a). Amplification reactions were carried out under the following thermocycling conditions: 95°C for 3 min, 30 cycles of 95°C for 30 s, 55°C for 30 s, 72°C for 30 s, with a final extension at 72°C for 5 min. PCR amplicons were confirmed by electrophoresis with a 1% (w/v) agarose gel and then purified by AMPure XP beads (Beckman Coulter) prior to the index PCR. Nextera XT Index was incorporated into each of the individual samples during PCR. The thermal cycling program included a first denaturation step at 95°C for 3 min, followed by 8 cycles of denaturation at 95°C for 30 s, annealing at 55°C for 30 s, elongation at 72°C for 30 s, with a final extension at 72°C for 5 min. Purified amplicons were submitted to KAUST Genomic Core Lab^1^ for unidirectional sequencing reads on an Illumina MiSeq platform.

### Statistical and co-occurrence network analyses

The sequence analysis of 16S rRNA gene amplicons was performed in QIIME2 using the DADA2 package. Trimmed sequences were dereplicated, denoised, and merged, and chimeras were removed. The resulting 16S rRNA gene Amplicon Sequence Variants (ASVs) were taxonomically classified *via* the SILVA ACT: Alignment, Classification and Tree Service^2^ online server (Pruesse et al., 2012). Alpha diversity estimates were generated by using the Shannon, Simpson’s, Chao1, and Evenness indexes with statistical significance determined by ANOVA in Calypso (Zakrzewski et al., 2017). The correlation of environmental parameters with the microbial community composition of samples was determined with Redundancy Analysis (RDA) in Calypso (Zakrzewski et al., 2017). *p*-Value was provided for the significance of the grouping. Microbial taxa associated with specific environmental conditions were identified by a Linear Discriminant Analysis Effect Size (LefSe) approach and plotted as a cladogram. This comparison used an all-against-all approach with cut-off values of 0.05 for the Kruskal-Wallis alpha and 2.0 for the Linear Discriminant Analysis (LDA). Additional insights regarding the significant enrichment of the microbial taxa identified in LefSe were visualized in box plots through rank test analysis by Kruskal-Wallis test.

A correlation network, i.e., co-occurrence network, was established to identify clusters of strongly associated microbial taxa. The nodes indicate the orders, while the edges, which are connecting the nodes, represent correlations between orders. In this analysis, we included the microbial orders with a relative abundance >1% in at least five of the 19 samples under study. The R package NetCoMi (Peschel et al., 2021) was used to build a co-occurrence network based on SparCC estimated correlation values (Friedman and Alm, 2012). Edges were retained in the final network if the Benjamini–Hochberg adjusted *p*–values were below a given threshold, FDR < 0.05.

### Near-full-length 16S rRNA gene amplification, sequencing, and analysis through EMIRGE

Universal primer pair 9F-1406R was used to amplify the near-full-length 16S rRNA from the total DNA extracted from Ay311 and Ay323 (for the DRY group), Ay312 and Ay313 (for the WET group). The PCR reaction mixture contained 10 ng of total DNA, 1x Takara Ex Taq buffer with MgCl_2_ (10x; Takara Bio Inc., Tokyo, Japan), primers 300 nM, BSA (Roche Life Science, Basel, Switzerland) 1 mg mL^−1^, dNTP mix 200 μM, Takara Ex Taq Polymerase 0.5 U, and water (Lichrosolv^®^; Merck, Darmstadt, Germany) up to a total volume of 50 μl. After shearing PCR amplicons using restriction endonucleases, libraries were prepared using NEB Next Ultra II FS DNA Library Prep Kit (New England Biolabs; Ghezzi et al., 2021b). Paired-end Illumina sequencing was performed at the Core Facility Molecular Biology of the Medical University of Graz (Austria). Raw reads were trimmed, and quality filtered using an in-house Galaxy set-up (Klymiuk et al., 2016), which included the algorithm Expectation Maximization Iterative Reconstruction of Genes from the Environment (EMIRGE), to carry out the reconstruction of (near) full-length 16S rRNA genes (approximately 1,400 bp long) from Illumina metagenomic sequences (Miller et al., 2011, 2013; Ghezzi et al., 2021b). We utilized the script EMIRGE_amplicon.py that allows the reconstruction of complete 16S rRNA genes from PCR amplicon sequencing data (Miller et al., 2013). Specifically, EMIRGE was run for 120 iterations with default parameters designed to merge reconstructed 16S rRNA genes if candidate consensus sequences shared ≥97% sequence identity in any given iteration (Miller et al., 2011, 2013). Reconstructed near-full-length 16S rRNA sequences were clustered into Operational Taxonomic Units (OTUs) at 97% identity to remove similar sequences. Chimeras were identified and removed with Uchime2 v11 (Edgar et al., 2011).

### Phylogenetic analysis of the EMIRGE reconstructed near-full-length 16S rRNA genes

Phylogenetic trees were constructed using the OTU sequences deriving from EMIRGE assembly or the ASVs obtained with DADA2. For each ASV/OTU sequence included in the trees, the most closely related sequences retrieved from the GenBank database (Best BLAST Hits) were included in the phylogenetic analyses. All the sequences (ASV/OTUs and reference sequences) were aligned with ClustalW and used to construct phylogenetic trees based on neighbor-joining clustering method using MEGAX with bootstrap values of 1,000 (Kumar et al., 2018).

## Results

### Environmental and geochemical analyses of the cave samples

All the analyzed samples were collected from cave zones showing stable temperatures ranging between 13°C and 15°C. The samples were subjected to several analyses to obtain the geochemical parameters reported in Supplementary Table S1. Based on XRD mineralogical composition, SiO_2_ dominated in 16 out of 19 samples, whereas three samples (Ay307, Ay308, and Ay315) were also composed of sulfates like gypsum and alunite (Supplementary Table S1). In addition, the XRF analysis of those samples providing enough material for this analytical method, showed that samples of quartzite, amorphous silica, and speleothems from cave walls, floor, and ceiling contained very low amounts of iron. This element was more abundant in the WATER samples Ay314 and Ay316. The latter also revealed the highest amount of aluminum detected among all samples. Barium was present in various cave samples independently from the cave niche.

### Sequencing results, clustering, and diversity indexes

A total of 5,244,569 raw reads were generated through Illumina sequencing and corresponded to 5,794 ASVs, with a minimum of 74 ASVs (Ay315) and a maximum of 869 ASVs per sample (Ay317; Supplementary Table S1).

**Figure 2:**
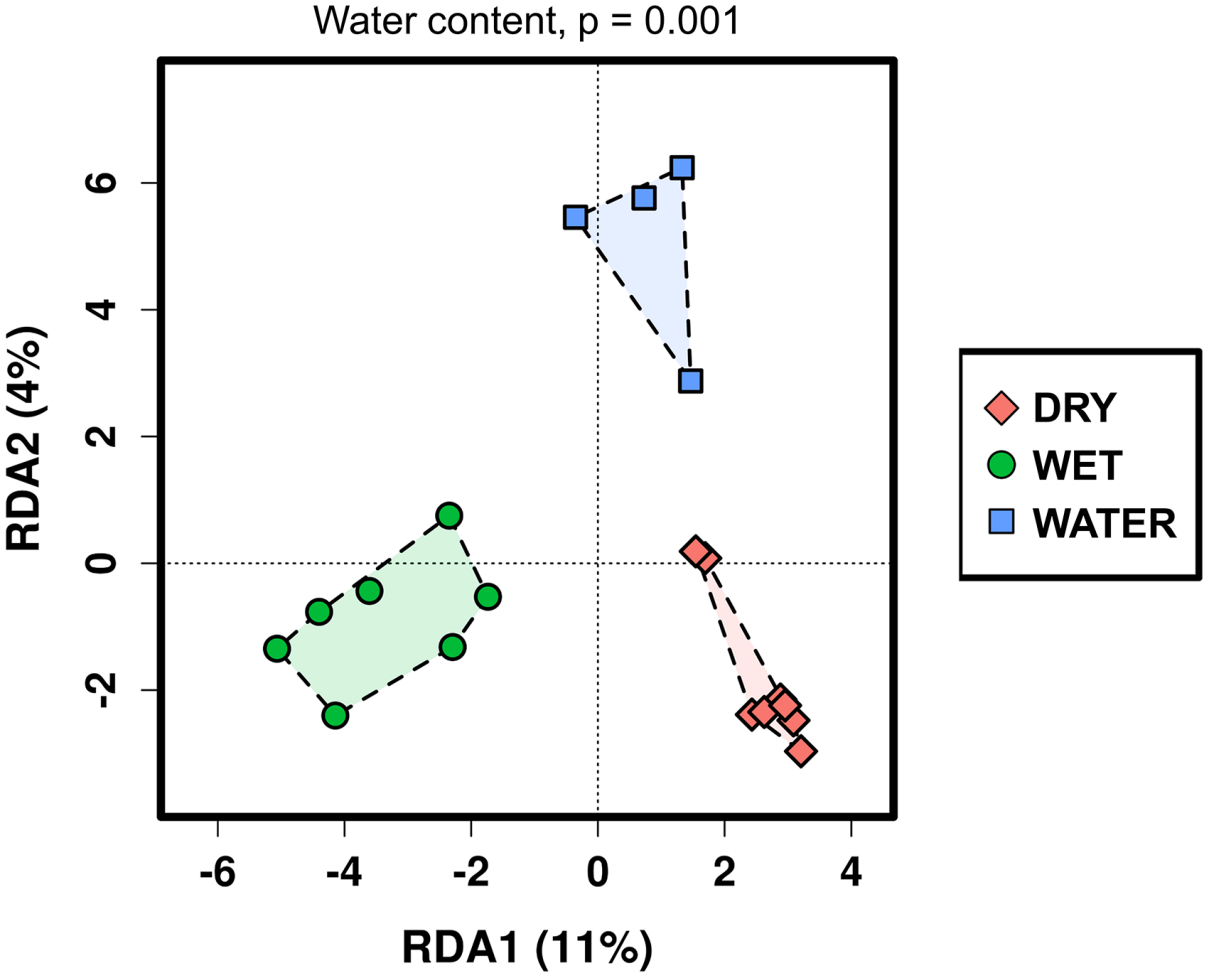
Redundancy analysis (RDA) of the Imawarì Yeuta microbial communities based on the water content of the cave samples. The value of *p* = 0.001 resulting from RDA analysis considering the water content as grouping parameter is indicated above the graph. The *p*-values of the other environmental parameters that were considered in the RDA analysis but did not result significant, are reported in Supplementary Table S2.

The relationship between the microbial communities’ composition and the available cave samples’ characteristics was analyzed using redundancy analysis (RDA; Figure 2). Water content, pH value, cave niche/location, major mineral composition, and sample color (indicated in Supplementary Table S1) were used as grouping parameters. As a result, RDA analysis distinguished three groups of samples corresponding to the three water content conditions (*p* = 0.001), whereas the effects of the other environmental and geochemical parameters were not significant (Supplementary Table S2). Based on the water content, the three groups were named WATER (water content >89%), WET (water content between 4% and 20%), and DRY (water content <1%). Regarding the cave collection sites, the WATER group included only samples collected from stagnant water ponds (with patinas and mats floating on the water surface). Conversely, both the WET and DRY groups included different sample types (orthoquartzite, sediments, and speleothems) deriving from different cave locations, i.e., the walls, the ceiling, and the floor. However, all the samples within the WET group shared the proximity of their collection site to small water flows (for those samples collected from the floor) or percolating water (for those samples collected from the cave wall).

**Figure 3:**
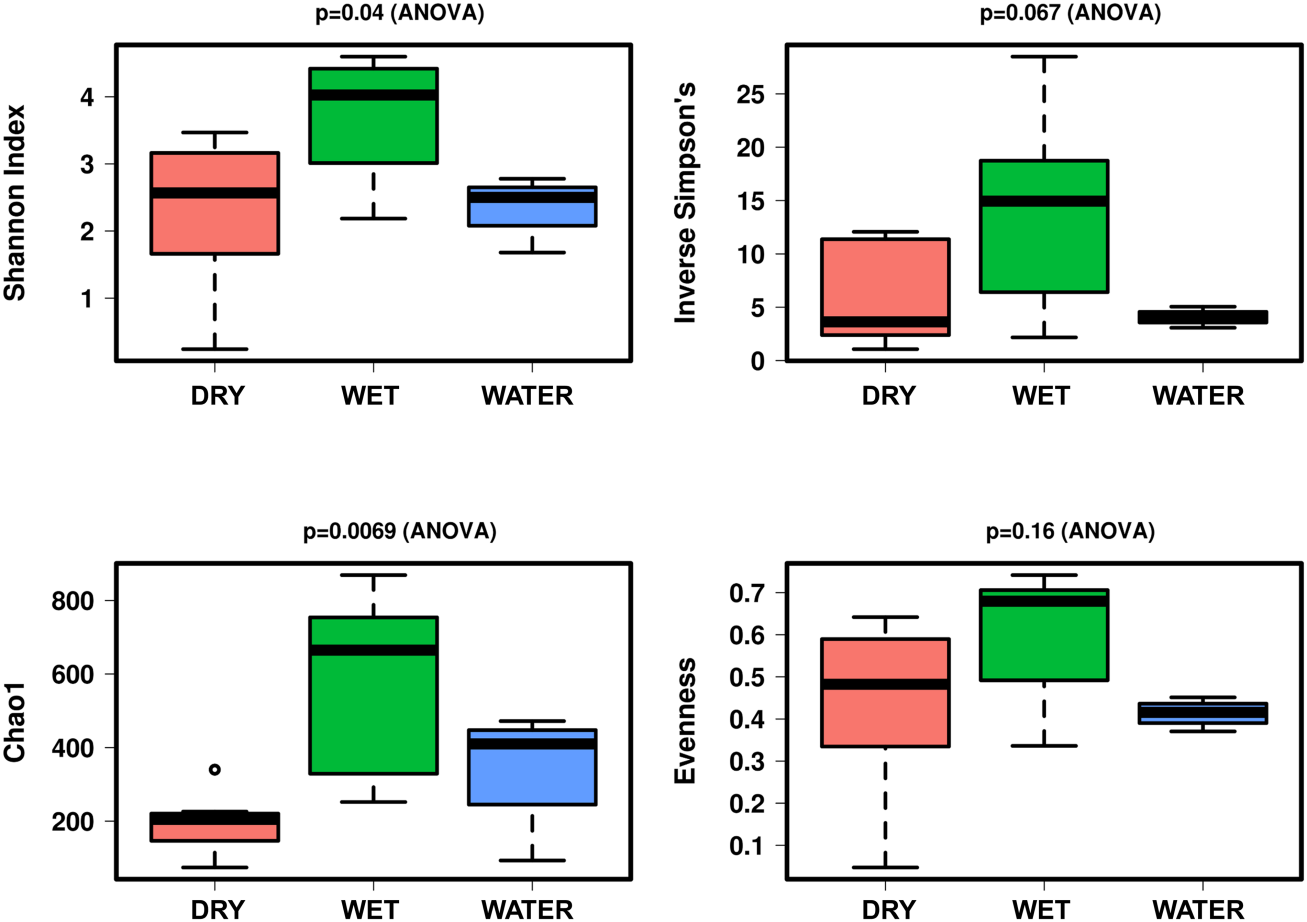
Boxplot of alpha diversity indices (Shannon, Simpson, Evenness, and Chao1) of the Imawarì Yeuta samples grouped based on water content. The *p*-value indicating the statistical significance of the analysis (calculated by one-way ANOVA) is indicated above each boxplot.

In terms of alpha diversity indices, the microbial communities of WET samples were significantly more diverse and richer as compared to those inhabiting WATER and DRY samples (Shannon: *p* = 0.04; Figure 3). These two groups showed similar diversity value, while DRY samples had the lowest Chao1 index (*p* = 0.0069).

### Microbial community composition

**Figure 4:**
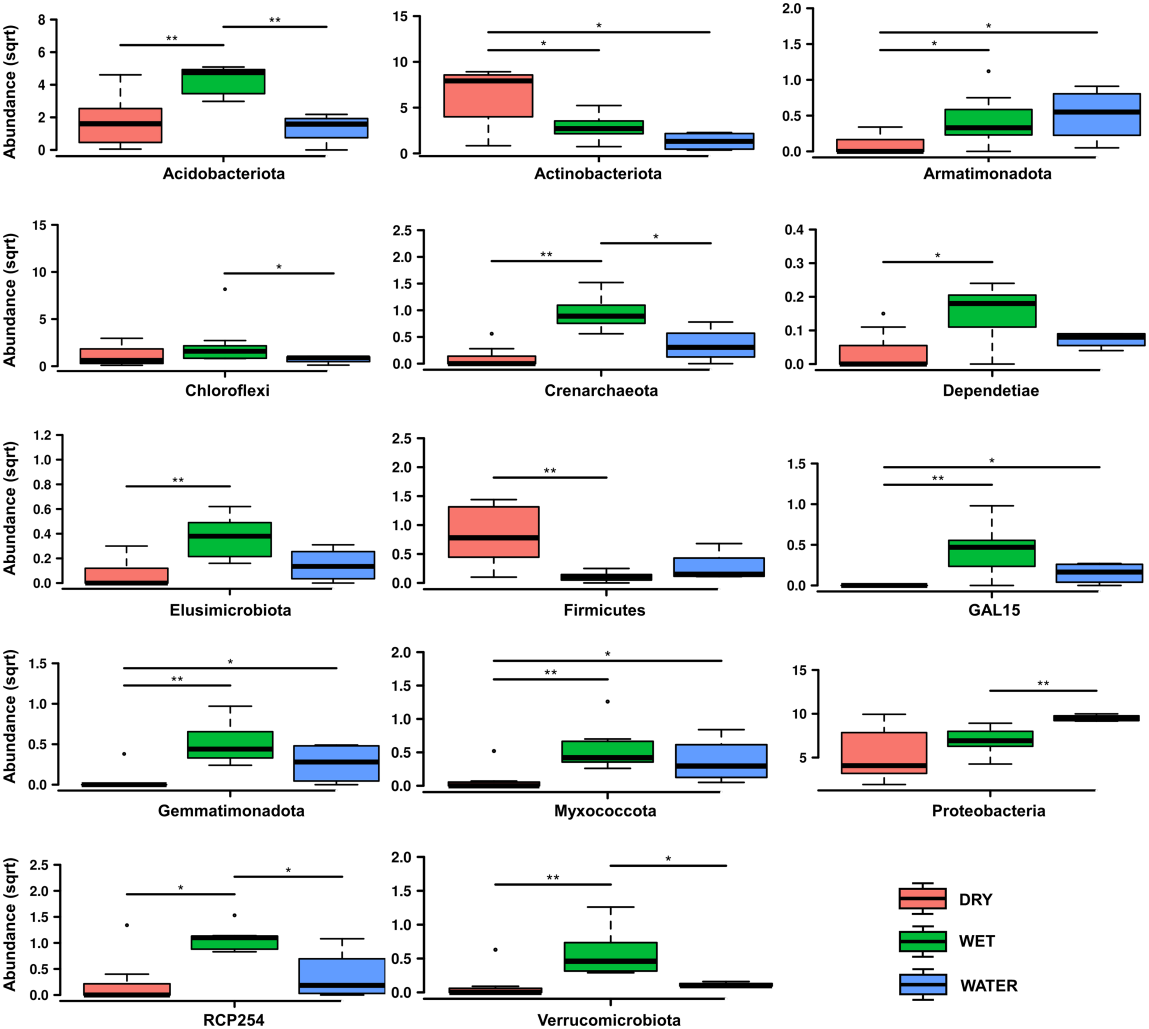
Rank test of significantly enriched microbial phyla in DRY, WET, and WATER sample groups. Abundance in the y axes stands for Relative abundance. The statistical significance is indicated by ^*^ and ^**^ that stand for *p* < 0.05 and *p* < 0.01, respectively. (sqrt) stands for square root transformation.

*Bacteria* dominated the microbial communities of all nineteen samples, while *Archaea* accounted for <1% in all samples except for the Ay312 and Ay330 belonging to the WET group, in which *Crenarchaeota* covered 2.3% and 1.5%, respectively. Rank tests analysis at phylum level showed significant differences in the relative abundance of *Acidobacteriota* (*p* = 0.0041), *Actinobacteriota* (*p* = 0.012), and *Proteobacteria* (*p* = 0.039; Figure 4), which characterized each of the three sample groups, i.e., WET, DRY, and WATER, respectively. In addition to *Acidobacteriota*, WET group was also significantly enriched with *Crenarchaeota* (*p* = 0.0017), RCP-254 (*p* = 0.015), *Verrucomicrobiota* (*p* = 0.0038), GAL15 (*p* = 0.0038), *Elusimicrobiota* (*p* = 0.0088), *Dependentiae* (*p* = 0.027), *Gemmatimonadota* (*p* = 0.006), and *Myxococcota* (*p* = 0.0084). In addition to *Actinobacteriota* and *Proteobacteria*, DRY and WATER were characterized by *Firmicutes* (*p* = 0.014) and *Armatimonadota* (*p* = 0.025; Figure 4), respectively.

**Figure 5:**
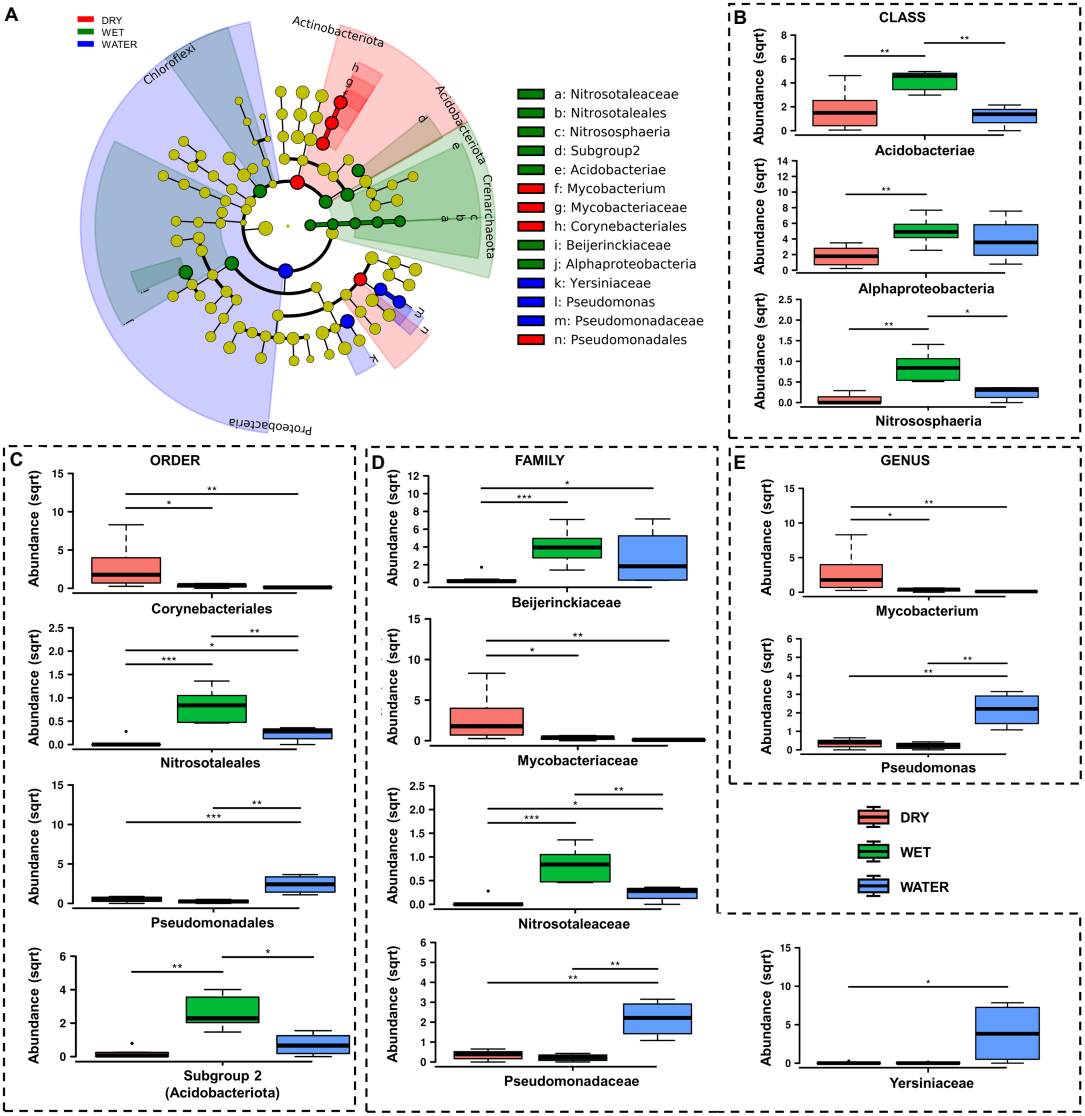
LefSe analysis of microbial communities from Imawarì Yeuta samples. **(A)** LefSe cladogram showing the microbial taxa (represented by the dots) with significant differences in the three groups. Red, green, and blue shades group different taxa, with the species classification at the level of phylum, class, order, family, and genus shown from the inside to the outside. The red, green, and blue nodes in the phylogenetic tree represent microbial taxa that play an important role in the DRY, WET, and WATER groups, respectively. Yellow nodes represent taxa with no significant difference. **(B–E)** Rank test results for classes, orders, families, and genera identified in LefSe analysis. Abundance in the y-axes stands for relative abundance. (sqrt) stands for square root transformation. The statistical significance is indicated by ^*^, ^**^, and ^***^ that stand for *p* < 0.05, *p* < 0.01, and *p* < 0.001, respectively.

At lower taxonomy levels, WATER was characterized by members of *Gammaproteobacteria* of *Pseudomonadaceae* family, and *Pseudomonas* genus (*p* < 0.0001), as well as members of *Yersiniaceae* family (*p* = 0.031) (Figures 5C–E). On the other hand DRY and WET groups were generally dominated by members of unclassified genera and/or families belonging to *Acidobacteriota* and *Alphaproteobacteria* in the WET samples and belonging to *Actinobacteriota* in the DRY samples (Figures 5A,B; Supplementary Figure S1). In particular, within these groups, WET group was mainly characterized by members of the class *Acidobacteriae* (*Acidobacteriota*, *p* = 0.0049), Subgroup 2 order (*Acidobacteriae*, *p* = 0.0011), unclassified *Acidobacteriota* (*p* < 0.001), *Beijerinckiaceae* family (*Alphaproteobacteria*, *p* = 0.001) and the archaeal *Nitrosotaleceae* family (*Nitrosotaleales*, *p* = 0.001) (Figures 5C,D). Microbial members enriched in DRY group belonged to *Acidimicrobiia* class (*Actinobacteriota*, *p* = 0.0049) of *Corynebacteriales* order (*Actinobacteria*, *p* = 0.005), *Mycobacteriaceae* family, and *Mycobacterium* genus (*p* = 0.024; Figures 5C–E).

### Dissecting community structure *via* co-occurrence network analysis

**Figure 6:**
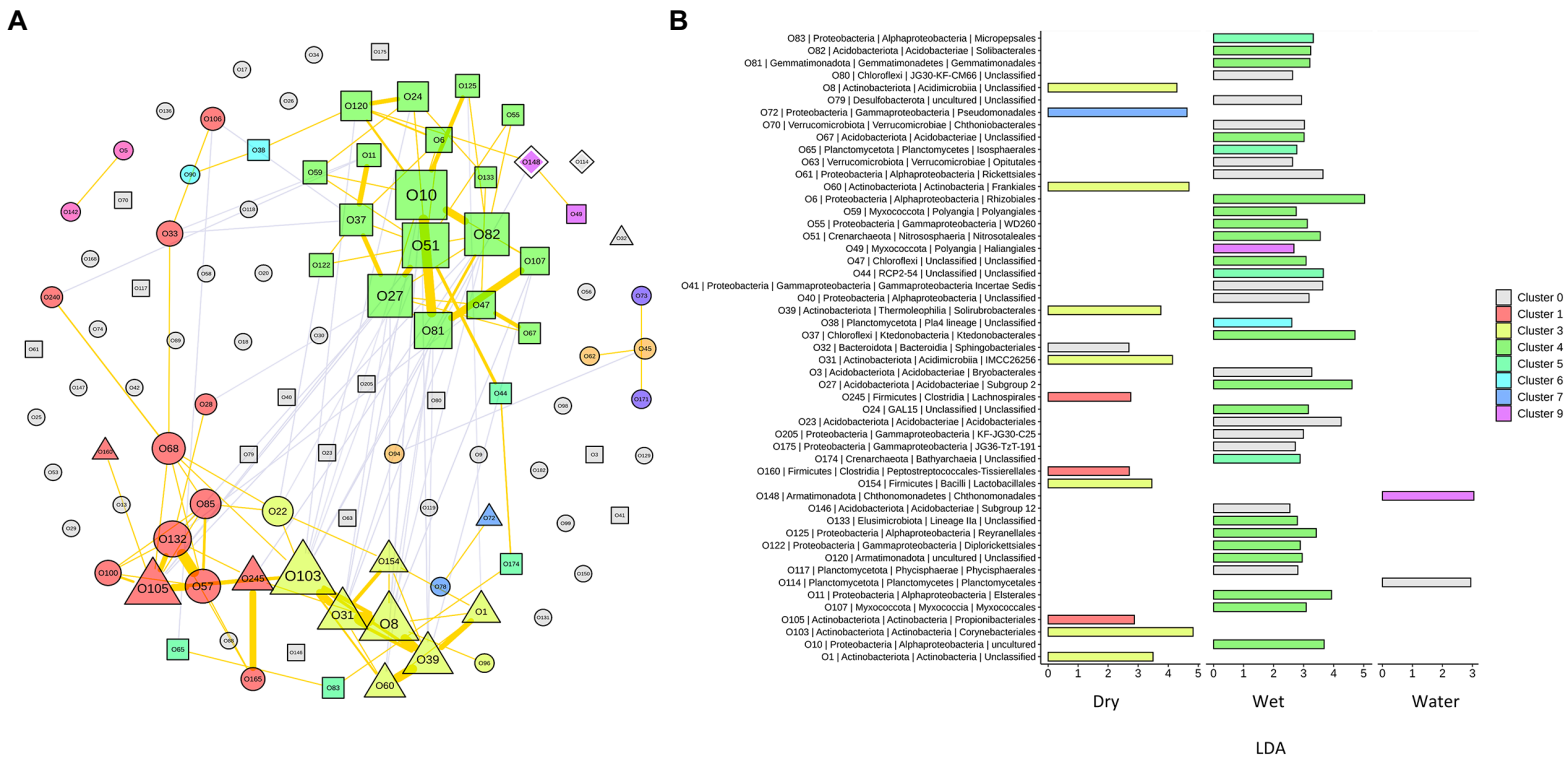
Network analysis of the microbial communities from Imawarì Yeuta cave. **(A)** Co-occurrence network of the most abundant orders (with relative abundance >1% in at least 5 samples) in the communities. Node codes (“O” followed by a number) correspond to the order/taxonomy shown in panel B, node color indicates the order assignment to each network cluster. Node size scales according to the node’s centrality. Node shapes correspond to the orders significantly enriched in DRY (triangle), WET (square), and WATER (diamond) groups. The circle shape indicates order that is not significantly enriched in any of the three sample groups. The yellow lines indicate positive interactions, and grey lines indicate negative interactions. The thickness of the lines is proportional to the correlation strength. **(B)** LDA scores of the orders included in the network analysis. The color shown in the legend indicates the cluster membership. C0 (in grey) indicates those orders that do not belong to any cluster.

To capture the relationships and interactions between microbial taxa present in Imawarì Yeuta samples, a co-occurrence network was built (Figure 6A). This analysis considered microbial diversity at the order level (Supplementary Table S3) since at lower taxonomic levels, i.e., family and genus, the data became too sparse to compute meaningful co-occurrence patterns. The resulting network showed that distinct co-occurrence relationships existed between members of the WET and DRY communities although a large proportion of taxa did not cluster together (modularity index of 0.34). WATER communities did not show any topologically distinct cluster.

In the resulting network, most of the co-occurrences were included in three clusters, i.e., Cluster1 (C1), C3, and C4, which comprised orders that characterized either the WET or DRY samples (Figure 6A). Specifically, cluster C4 included orders that were dominant (Subgroup 2 and *Solibacteriales*) and significantly enriched (*Rhizobiales*, *Nitrosotaleales*, and *Ktedonobacterales* with LDA > 3) in the only WET samples (Figure 6B). Clusters C1 and C3 included most of the orders that were enriched in DRY samples. C3 clustered members of the orders *Corynebacteriales*, *Frankiales*, *Solirubrobacterales,* and other three uncharacterized/unclassified orders of *Acidimicrobiia* and *Actinobacteria* classes (Figure 6B). Cluster C1 comprised members of the orders *Propionibacteriales*, *Peptostreptococcales*, and *Lachnospirales*. Therefore, co-occurrences in C1 and C3 indicated relationships between orders from DRY samples belonging to *Actinobacteriota* and *Firmicutes* phyla. Conversely, C4 included orders from WET samples that belonged to various phyla; those having the highest LDA score were *Acidobacteriota*, *Proteobacteria (Alphaproteobacteria), Chloroflexi*, and *Crenarchaeota* (Figure 6B).

### Phylogenetic analyses of the microbial members dominating each sample group

We further analyzed the phylogenetic relationship of the members belonging to the phyla characterizing each of the sample group WET (*Acidobacteriota*), DRY (*Actinobacteriota*), and WATER (*Proteobacteria,* specifically *Gammaproteobacteria*), with reference sequences from the NCBI database (Supplementary Figures S2–S4; Supplementary Table S4). In WATER samples, the ASVs belonging to *Gammaproteobacteria* showed high similarity (>99%) with reference sequences of isolated and cultured strains which are characterized at genus level (Supplementary Figure S2). Among these, the ASVs belonging to *Burkholderiales* were mainly affiliated to the genera *Delftia*, *Janthinobacterium*, *Nitrosospira*, and *Nitrosovibrio* and share high similarity with clones and sequences retrieved from volcanic deposits, lava caves, marine and lake environments, FACE soils, and other caves (Supplementary Table S4). Among these, the *Janthinobacterium*-affiliated ASV4662 shared 100% of similarity with clones retrieved from the quartzite Roraima Sur Cave (Venezuela) and the stripe karst (marble within quartz-dominated schists) Raspberry Rising Cave (Glacier National Park, Canada) (Supplementary Table S4). Other ASVs abundant in Imawarì Yeuta water samples were highly affiliated (>99%) with *Pseudomonas*, *Stenotrophomonas*, and *Acinetobacter* genera, and to different genera of the *Enterobacterales* order, including *Erwinia*, *Serratia*, and *Enterobacter*, retrieved from acidic environments, forest soils, and tropical marine locations. The *Serratia*-affiliated ASV513 and the *Pseudomonas*-affiliated ASV1934 shared 100% of similarity with sequences retrieved from a Hungarian karst cave and Lascaux Cave (France), respectively (Supplementary Table S4).

Different to WATER samples, the ASVs belonging to the dominant taxa in the DRY and WET groups, i.e., *Actinobacteriota* and *Acidobacteriota*, generally showed low similarity (<98%) with sequences from strains that are taxonomically characterized at family and genus levels (Supplementary Table S4). The only exceptions were the ASV304 and ASV322 belonging to *Mycobacterium* genus. In the light of the novelty of the microorganisms characterizing the WET and DRY samples, we decided to deeply analyze the dominant taxa of these sample groups. Therefore, we reconstructed and studied the near-full-length sequences (OTUs) of the 16S rRNA gene of the dominant groups of WET and DRY samples, i.e., *Acidobacteriota* and *Actinobacteriota*, by carrying out EMIRGE analysis. We performed this analysis on two representative samples from each group, i.e., Ay311 and Ay323 for the DRY group, Ay312 and Ay313 for the WET group. The OTUs were further used to construct phylogenetic trees together with the reference sequences from the NCBI database (Supplementary Figures S3, S4). As a result, the most abundant OTUs affiliated to *Acidobacteriota* from WET samples formed two distinct clades, one including members belonging to the Subgroup 2 order and the other including members of the *Acidobacteriales* order. They shared their highest nucleotide similarity (97%) with reference sequences of uncultured strains retrieved from quarzitic soils and samples collected within different cave systems including lava tubes and the quartzite Roraima Sur Cave (Supplementary Figure S3). The most abundant *Actinobacteriota*-related OTUs in DRY samples included many members affiliated to *Mycobacterium* genus, although the sequence identity with reference strains was below 98%, suggesting a certain degree of taxonomy separation from the database sequences (at species or even genus level; Yarza et al., 2014). Among the closest uncultured reference sequences, we identified the highest nucleotide similarities with many actinobacterial clones retrieved from other cave environments including Roraima Sur Cave, Lower Kane Cave (Wyoming, USA), and lava tube walls, even though the percentage identity was generally low (94%–97%; Supplementary Figure S4). In line with this, our OTUs formed clades in the tree that were distinct from both cultured and uncultured references, except for OTU1 that clustered with clone sequences retrieved from Roraima Sur Cave (98%–99% of nucleotide sequence identity). The taxonomy affiliation of this OTU and its branch was not definable as the closest reference strains only shared 93%–94% of nucleotide identity and belonged to different genera including *Goodfellowiella*, *Pseudonocardia*, *Rhodococcus*, and *Streptoalloteichus*. In addition to the similarity with sequences from cave environments, many DRY and WET OTUs shared high similarities with uncultured bacteria retrieved from other oligotrophic and silica-rich environments that however were not subterranean such as FACE soils, ferralsol, and bamboo forest soils (Supplementary Figures S3, S4).

## Discussion

This work reports the microbiome of a quartzite cave by describing the composition and structure of microbial communities inhabiting the different niches of Imawarì Yeuta. Quartzite caves in tepuis represent peculiar environments for microbial growth as, in addition to the absence of light, the orthoquartzite rock has a low buffering capacity. The deep zones of this cave are also characterized by organic carbon limitation. Possible sources of nutrients derive from the water leakage or condensation of water moisture from the surroundings (Sauro et al., 2018). These infiltration/interstitial waters are transparent and show extremely low conductivity values (close to distilled water) with contents of organic acids and dissolved carbon always below the detection limits (Mecchia et al., 2014). In this work, we found that, among the environmental parameters analyzed, only water content/presence significantly influenced the microbial communities’ structure and composition within Imawarì Yeuta (Figure 2). In a previous study, Barton et al. (2014), suggested the major impact of pH on the microbial communities present in three rocky samples from the quartzite Roraima Sur Cave (RSC). These samples were collected at different distances from the cave entrance and were characterized by acidic pH values ranging between 4.9 and 5.6. In our work, we did not find the pH value to significantly correlate with the microbiome of Imawarì Yeuta (Supplementary Table S2), although the pH values of the samples we analyzed were similar to those from Roraima Sur Cave. Moreover, no significant correlation was found between the microbial community structure and the other parameters we could analyze, including the presence of specific minerals and the cave location (floor, wall, ceiling). Conversely, previous studies reported a significant correlation between the microbial composition and the cave niche (defined as air, rock, sediment, or water) or the mineral substrate present in limestone and dolomite caves (Alonso et al., 2018; Dhami et al., 2018; Zhu et al., 2019). These correlations were mostly associated with the different nutrient levels related to the analyzed cave niches and their different origins (i.e., rocks were related to autochthonous origin from the cave, while sediments were allochthonous and brought into caves from the outside). The absence of significant correlation between the diversity of the microbial communities and the cave niche/location we report here, might be due to the fact that, differently from the previous cave studies, all the niches we analyzed in Imawarì Yeuta are under the same nutritional (oligotrophic) conditions. Some indications on the poor nutrient conditions of tepuis’ caves are reported in works by Barton et al. (2014) and Mecchia et al. (2014). On the other hand, our results are in line with other studies that showed the water content to be the main driving force of the microbial communities’ composition in various environments such as agricultural and grassland soils, surface litter, and deserts (Stres et al., 2008; Manzoni et al., 2012; Li et al., 2021). However, some environmental parameters could not be included in our RDA analysis, such as the minor elements and the silica amorphization stage, as they were available only for a few samples. Our previous studies suggested a relation between the silica amorphization phase and the microbial diversity in Imawarì Yeuta (Sauro et al., 2018; Ghezzi et al., 2021b). The lack of knowledge on the silica amorphization stage of most of the samples did not allow the evaluation of this environmental factor in the correlation analysis, although this could greatly influence microbial communities’ structures and compositions. In this regard, based on full 16S rRNA gene similarity analysis, the silica amorphization stage seemed to have a strong impact on *Ktedonobacterales* members (Ghezzi et al., 2021b). Indeed, these strains were more phylogenetically related when we compared samples with similar silica amorphization stages rather than samples collected from proximal locations (Ghezzi et al., 2021b).

Based on the correlation with the water content, the samples of Imawarì Yeuta were divided in the three groups DRY, WET, and WATER (Figure 2). The samples with intermediate water content levels (WET) presented higher richness and diversity as compared to WATER and DRY samples (Figure 3). This can be related to previous findings that reported an increase of microbial richness depending on the moisture in soil microcosms and the promotion of rare bacterial species in the presence of intermediate water content (Xu et al., 2017; Bickel and Or, 2020). The higher diversity and richness shown by the WET samples from Imawarì Yeuta might be due in part to the microelements provided by the water that enters this cave mostly by dripping through cracks from the overlying rocks. The water in this cave has been reported to be extremely deficient in both organic carbon and inorganic dissolved chemicals (Mecchia et al., 2014). However, this low amount of nutrients, including oxygen and other gases, can greatly impact microbial diversity in oligotrophic and aphotic environments (Zhou et al., 2002; Stres et al., 2008; Hershey et al., 2018; Ghezzi et al., 2021b). Unclassified genera, families, and orders belonging to several phyla (e.g., *Elusimicrobiota*, *Gemmatimonadota*, *Dependetiae*) were significantly enriched in WET samples. Specifically, this group was dominated by members of Subgroup 2 and *Solibacterales* of *Acidobacteriota* phylum, and members of *Beijerinckiaceae* family of *Alphaproteobacteria* class (Figures 5C,D). Similarly, previous studies demonstrated that the rewetting of arid soils favored the increase of the microbial diversity together with the establishment of *Acidobacteriota* and the decrease of *Actinobacteriota* (Barnard et al., 2015; Schimel, 2018; She et al., 2018). In line with this, we found members of *Actinobacteriota* to be enriched in DRY samples that also showed the lowest values of microbial diversity and richness (Figures 3, 4–5). *Actinobacteriota*- and *Firmicutes*-affiliated microbial taxa associated with the DRY samples, such as the actinobacterial members of *Corynebacteriales* order and *Pseudonocardiaceae* family, have been previously found to dominate low-diverse microbial communities and typically inhabit arid soils due to their resistance to adverse environmental conditions including nutrient shortage, acidic pH, and scarce availability of water (Mohammadipanah and Wink, 2016; Rego et al., 2019).

The dominant taxa of the DRY and WET groups also showed a correlation at order level in the co-occurrence network analysis (Figure 6A). This indicates ecological interactions occurring among the microbial taxa as well as their associations to the possible differences in physicochemical and nutritional variables present in the DRY and WET groups. The network analysis grouped the orders characterizing the DRY samples that only belonged to *Actinobacteriota* and *Firmicutes*, in the two clusters C1 and C3 (Figure 6B). A separate single cluster (C4) exclusively included orders characterizing the WET samples; these orders belonged to various phyla. This indicates that in WET samples ecological interactions occur among microbial strains that are more heterogeneous from a taxonomy point of view (as compared to the DRY samples), in turn suggesting that the dominant microbial taxa contribute to different functions in each sample group or cave niche (Mandakovic et al., 2018; Ma et al., 2021). Furthermore, as the network clustering was generated based on positive correlations, the inclusion in one single cluster (C4) of almost all the dominant orders characterizing WET samples indicates that these taxa mostly rely on cooperative relationships rather than on competitive ones. Microbial cooperation is a possible strategy to survive and persist under poor nutrients and adverse conditions. Promotion effects among microbes were previously detected through network analysis of microbial communities from other oligotrophic caves (Ma et al., 2021). On the other hand, orders dominant in WATER samples did not generate any topologically distinct clusters. This might be due to the reduced number of samples and/or to the higher heterogeneity of the WATER samples as compared to WET and DRY in terms of microbial community composition.

Generally, we found *Pseudomonas* and, more generally, *Gammaproteobacteria* to be enriched in Imawarì Yeuta WATER samples (Figure 5E; Supplementary Figure S1). This finds analogies with other caves that were described hosting high abundance of *Pseudomonadales* and former *Betaproteobacteria* in cave waters (Shabarova and Pernthaler, 2010; Shabarova et al., 2013; Brannen-Donnelly and Engel, 2015; Zhu et al., 2019). In particular, Zhu et al. (2019) assessed that the moisture level in karst caves promotes the establishment of *Proteobacteria* species and represents the most important factor affecting the microbial community composition.

All the dominant microbial taxa we identified in this study to be significantly enriched in WET, DRY, and WATER samples have been described to include genera/species involved in biomineralization processes and/or rock weathering. In particular, several *Acidobacteriota* members of the WET-characterizing Subgroup 2 order have been associated with mineral-rich acidic and quartz-based sandy soils. They have been described to be involved in mineral weathering and to be capable of slowing down their metabolism to persist under starving conditions on quartzite substrates (Jones et al., 2009; Ward et al., 2009; Nishiyama et al., 2012; Wakelin et al., 2012). Members of *Alphaproteobacteria* of Imawarì Yeuta WET samples, mostly belonging to the nitrogen-fixing *Rhizobiales*, were significantly abundant in sediment or rock samples from other karst caves (Zhu et al., 2019). Furthermore, metabolically active *Rhizobiales* have also been found underneath desertic quartzitic rocks suggesting their contribution in providing primary reactions to sustain the development of other microbial groups (Van Goethem et al., 2017). Ammonia-oxidizers of the family *Nitrosotaleaceae* (*Crenarchaeota*) were also significantly enriched in WET samples. Although they are low in abundance, they might contribute to the nitrogen cycle in Imawarì Yeuta as suggested in other caves (Mihajlovski et al., 2019). Cave-dwelling *Actinobacteriota* belonging to DRY-abundant *Corynebacteriales* and, at lower extent, *Solirubrobacterales* and *Pseudonocardiales* orders were described to be involved in both biomineralization and rock weathering in volcanic and limestone cave rocks (Cuezva et al., 2012; Cockell et al., 2013) and were found to be associated with the most advanced stages of silica-amorphization and formation of coralloid speleothems in Imawarì Yeuta (Ghezzi et al., 2021b). RNA-based cave studies suggested *Actinobacteriota* to be among the most abundant metabolically active microbial group in caves and to be capable to exploit complex metabolic pathways to switch their metabolism according to the settings of the environment they live in (Gonzalez-Pimentel et al., 2018; Paun et al., 2019). Biomineralization activities were also observed in cave samples enriched in *Gammaproteobacteria* including WATER-characterizing *Pseudomonas*, *Serratia*, and *Janthinobacterium* genera (Rusznyák et al., 2012; Carmichael et al., 2013). In particular, iron biomineralization processes associated to these genera have been observed in ferromanganese deposits from carbonate caves (Rusznyák et al., 2012). Interestingly, noticeable iron amounts were detected in Imawarì Yeuta cave waters.

The most abundant ASVs belonging to WATER samples revealed sequence identity >99% with strains that are classified up to genus level (in the GenBank database) and/or that have been isolated from different environments (Supplementary Table S4). On the other hand, both the ASVs and OTUs characterizing the DRY and WET samples shared relatively low similarities with sequences in the database, and the Best BLAST Hits corresponded to unclassified sequences retrieved from other caves such as the quartzite Roraima Sur Cave, lava tubes, the limestone Lascaux Cave and Raspberry Rising Cave and oligotrophic/extreme locations, like Antarctic environments and deserts (Supplementary Table S4; Supplementary Figures S3, S4). The high novelty of microbial strains retrieved from the WET and DRY samples is probably due to the fact that the microbiology of silica-rich rocks/minerals and speleothems associated with quartzite cave environments is still mostly unknown. Conversely, microbes from Imawarì Yeuta WATER share similarities with strains retrieved from other fresh and river waters, acidic environments, and cave samples (Supplementary Table S2). This is probably due to the fact that, in Imawarì Yeuta, the surface environments above the cave have more influence, even if still limited compared to other ecosystems, on waters infiltrating in the cave rather than on the silica rocks/minerals. Furthermore, quartzite rocks/minerals in Imawarì Yeuta cave probably impose unique selective pressures leading to a specific microbial communities’ evolution and selection (i.e. microbial endemism).

Additional environmental sources of microbial strains taxonomically affiliated with those from Imawarì Yeuta are FACE soil, ferralsol, and bamboo forest soil (Supplementary Table S4). FACE (Free-Air CO_2_ and O_3_ Enrichment) soil shares the high partial pressure of CO_2_ in the atmosphere with caves (Dunbar et al., 2012; Houillon et al., 2017), while ferralsol and bamboo forest soil are well known to contain/present high amounts of silica and quartz (Collin et al., 2012; Jordanova, 2017). In parallel with this, on one hand, the use of atmospheric gases, like CO_2_ and CO, has been recently described as one of the main microbial strategies to thrive under low organic carbon conditions (Ji et al., 2017; Ortiz et al., 2021; Ghezzi et al., 2021b), on the other hand, quartzite rocks have very low pH buffering capacities (Barton et al., 2014). In line with the latter, WET-derived archaeal ASVs shared 100% of 16S rRNA sequence similarity with clones retrieved not only from silica-rich forest sandy soil and rice paddy soil (Okuda and Takahashi, 1962; Chen et al., 2015) but also from acid mine drainage and gold mines that are characterized by acidic pH values (Kuang et al., 2013; Supplementary Table S4). Taken together, our results indicate that, within the Imawarì Yeuta cave, water content/presence greatly influences the microbial ecology, while when comparative and phylogenetic analyses are performed, oligotrophic conditions, high CO_2_/CO partial pressures, and silica richness (in relation with the low pH) promote the dominance of specific microbial species.

In conclusion, this work provides a first global characterization of the biodiversity present in the quartzite Imawarì Yeuta cave, offering insights into some of the major environmental factors shaping the microbial communities thriving in oligotrophic quartzite caves. The water availability was found to greatly contribute to the microbial community structure and composition within Imawarì Yeuta, probably in association with the role of water in energy and nutrient supply. Accordingly, microbial taxa established specific co-occurrence patterns within clusters partially linked to water content. Conducting a thorough phylogenetic analysis of the dominant microbial members distinctive of the Imawarì Yeuta environment, we found their affiliation with microbial members retrieved from environments characterized by silica dominance in the rock substrate and/or CO_2_/CO atmospheric enrichment, suggesting these two being the main factors underlying the microbial diversity in association with the geochemistry of silica/quartz, the low pH value and possible alternative survival strategies. Future functional studies will investigate the genetic and metabolic aspects that contribute to the microbial interaction with the silica substrate and the microbial development in this unique oligotrophic quartzite cave.

## Data availability statement

The data presented in the study are deposited in the NCBI repository (https://www.ncbi.nlm.nih.gov/), accession number PRJNA796549.

## Author contributions

DG and MC designed the work, interpreted the data, and wrote the manuscript. FS, JW, and FV collected the samples. DG extracted the DNA from the cave samples. PH performed and financed the Illumina MiSeq run (V4-V5 region of the 16S rRNA gene amplicon) and conducted the initial quality check and filtering of the sequencing data. FS performed the geochemical analyses, made the cave map figure, and helped with the writing and interpretation of the manuscript results. DG, AF, and LF performed the bioinformatic analyses (both the 16S rRNA V4-V5 region and the near-full-length 16S rRNA gene sequencing data). AF made the network analysis and the corresponding figure, and helped in the network analysis text writing and interpretation. MC and FS supervised the work and contributed to funding acquisition. All authors contributed to the article and approved the submitted version.

## Funding

We acknowledge the Rector Francesco Ubertini, the Vice-Rector for Research A. Rotolo, and the Governing Academic Bodies of the University of Bologna (UNIBO) for the financial support of the research project and the PhD scholarship for DG. The research activities were also partly supported by RFO UNIBO grants. Funding for EMIRGE analysis was provided by Europlanet 2020 17-EPN3-026 grant. Europlanet 2020 RI has received funding from the European Union’s Horizon 2020 research and innovation program under grant agreement No 654208.

## Conflict of interest

The authors declare that the research was conducted in the absence of any commercial or financial relationships that could be construed as a potential conflict of interest.

## Publisher’s note

All claims expressed in this article are solely those of the authors and do not necessarily represent those of their affiliated organizations, or those of the publisher, the editors and the reviewers. Any product that may be evaluated in this article, or claim that may be made by its manufacturer, is not guaranteed or endorsed by the publisher.

## Footnotes

^1^ https://corelabs.kaust.edu.sa/

^2^ http://www.arb-silva.de/aligner
