# Supplementary material for: Insights into the microbial life in silica-rich subterranean environments: microbial communities and ecological interactions in an orthoquartzite cave (Imawarì Yeuta, Auyan Tepui, Venezuela)
Source: Front Microbiol. 2022 Sep 23;13:930302. doi: 10.3389/fmicb.2022.930302 (PMC9537377; doi:10.3389/fmicb.2022.930302)
Supplement: Supplementary file 1 [file Data_Sheet_1.pdf]

Supplementary Material

Supplementary Table S1. Geochemical and mineralogical characteristics, Illumina sequencing results, and description of the Imawarì Yeuta cave samples.

Sample name	Sample type	Description†	Cave location/niche	Water (%)	Mineral (XRD)§	SiO_2 (mg/L)	Al (µg/L)	Fe (µg/L)	K (mg/L)	Ba (µg/L)	Cl (mg/L)	SO_4 (mg/L)	NH_4 (mg/L)	Cu (µg/L)	Ca (µg/L)	Mg (µg/L)	pH	T (°C)	16S rRNA reads	ASVs	First study and sample name
Ay303	WATER	Iridescent violet slime floating on stagnant water ponds	Water pond	92.6	(floating) Opal-G	8.6	bdl	bdl	bdl	2.82	1.15	0.63	bdl	bdl	bdl	bdl	6.1	14.8	176330	472	WB (Sauro et al., 2018)
Ay314	WATER	Violet slime floating on water ponds	Water pond	95.1	-	8.87	bdl	19.7	bdl	6.5	0.69	0.66	bdl	bdl	bdl	bdl	5.1	14.8	464766	397	This work
Ay316	WATER	Black gours filled by percolating water with goethite	Water pond	89.0	(floating) Goethite	6.3	57	10.1	bdl	1.61	0.98	0.59	bdl	bdl	bdl	bdl	5	14.4	197791	423	This work
Ay325	WATER	Iridescent white patina on cave water	Water pond	96.1	-	8.6	bdl	bdl	bdl	3	1.1	0.6	bdl	bdl	bdl	bdl	6.1	14.8	386854	93	This work
**Sample name**	**Sample type**	**Description†**	**Cave location/niche**	**Water (%)**	**Mineral (XRD)§**	**SiO_2 (wt%)**	**Al_2O_3 (wt%)**	**Fe_2O_3 (wt%)**	**K_2O (wt%)**	**Ba (ppm)**	**Cl (ppm)**	**SO_4 (ppm)**	**NH_4 (ppm)**	**Cu (ppm)**	**Ca (wt%)**	**Mg (wt%)**	**pH**	**T (°C)**	**16S rRNA reads**	**ASVs**	**First study and sample name**
Ay301	WET	Whitish dots of amorphous silica on an orthoquartzite rock wall	Wall	9.9	Quartz	*	-	-	-	-	-	-	-	-	-	-	5	14.9	402695	665	This work
Ay302	WET	White paste of amorphous silica on an orthoquartzite rock wall	Wall	15.2	Quartz, Opal-G	93.9	1.6	0.7	0.05	37	52	295	75	88	0.01	0.1	5	14.9	160318	335	WL (Sauro et al., 2018)
Ay304	WET	Spots on a reddish orthoquartzite rock on the cave floor	Floor	18.5	Quartz	98	1.6	0.1	0	0	11	0	0	16.2	0	0	5	14.9	227736	725	QP (Ghezzi et al., 2021b)

Ay305	DRY	White shiny patinas on the orthoquartzite cave floor	Floor	1.0	Quartz, Opal-G	86.9	1.6	0.7	0.048	37	52	296	10	88.1	0	0.1	5	14.9	120041	205	SC (Ghezzi et al., 2021b)
Ay306	DRY	Black amorphous silica coralloid on the cave floor	Floor	0.8	Opal-G, Opal-A	93.9	0.8	0.4	0.012	76	151	171	471.4	23.7	0	0	5	14.9	401729	226	SA (Ghezzi et al., 2021b)
Ay307	DRY	Soft white deposits of sulphates on the orthoquartzite cave floor	Floor	0.7	Gypsum, Opal-G	*	-	-	-	-	-	*	-	-	-	-	5	14.5	430006	215	This work
Ay308	DRY	White powder of sulphates and amorphous silica on the cave floor	Floor	0.7	Gypsum, Opal-G	*	-	-	-	-	-	*	-	-	-	-	5	14.5	427594	340	This work
Ay309	DRY	Mold on coralloids on the cave roof (black)	Ceiling	0.2	Opal G	*	-	-	-	-	-	-	-	-	-	-	5	14.5	505692	156	This work
Ay311	DRY	Violet patina on the cave roof	Ceiling	0.5	Opal G	*	-	-	-	-	-	-	-	-	-	-	6	14.5	161414	137	This work
Ay312	WET	Yellow dots and agglomerates of quartz sand	Wall	6.9	Quartz	*	-	-	-	-	-	-	-	-	-	-	3.5	14.5	27947	252	This work
Ay313	WET	Black encrustation of amorphous silica on quartz sand	Wall	6.2	Quartz, Opal-G	98.48	0.93	0.15	0	0	0	0	0	13.5	0	0.02	3.5	14.9	220870	783	S (Sauro et al., 2018)
Ay315	DRY	White powder of alunite and gypsum deposit	Floor	0.1	Alunite, Gypsum, Opal G	*	*	-	*	-	-	*	-	-	-	-	6.5	14.5	269667	74	This work
Ay317	WET	Orthoquartzite wall (reddish)	Wall	7.6	Quartz	98	1.6	0.1	0	0	11	0	0	16.2	0	0.02	4	14.9	274724	869	Q (Sauro et al., 2018)
Ay323	DRY	Mold on silica coralloids on the cave roof (black)	Ceiling	0.1	Opal-G, Opal-A	82.4	1	0.57	0.04	107	77	274	2	21.5	0.02	0.08	5	14.5	365864	977	This work
Ay330	WET	Patina on weathered reddish orthoquartzite rock	Wall	5.6	Quartz, Opal-G	*	-	-	-	-	-	-	-	-	-	-	5	13	22531	323	This work

***** Major component from XRD and EDS mineral analysis
* Component detected in XRD and EDS mineral analysis
\- Not measured
bdl Below Detection Limit of ICP water analysis
§ Corresponds to major mineral composition, the major mineral component for each sample is underlined (it was used for RDA analysis)
† The main coloration of the sample is underlines (it was used for RDA analysis)

Supplementary Table S2. Results of the redundancy (RDA) analysis in Imawarì Yeuta cave.

Parameter	P-value
Cave niche/location	0.395
Sample colour	0.093
Water content	0.001**
pH	0.494
Major mineral composition	0.766

The symbol ** represents the significance ($p < 0.05$)

Supplementary Table S3. List of all the microbial orders considered in the network analysis shown in Figure 6.

order_id	Phlyum	Class	Order
O1	Actinobacteriota	Actinobacteria	Unclassified
O2	Acidobacteriota	Subgroup 22	Unclassified
O3	Acidobacteriota	Acidobacteriae	Bryobacterales
O4	Hydrogenedentes	Hydrogenedentia	Hydrogenedentiales
O5	Acidobacteriota	Vicinamibacteria	Vicinamibacterales
O6	Proteobacteria	Alphaproteobacteria	Rhizobiales
O7	NB1-j	Unclassified	Unclassified
O8	Actinobacteriota	Acidimicrobiia	Unclassified
O9	Planctomycetota	Planctomycetes	Gemmatales
O10	Proteobacteria	Alphaproteobacteria	uncultured
O11	Proteobacteria	Alphaproteobacteria	Elsterales
O12	Proteobacteria	Alphaproteobacteria	Rhodospirillales
O13	Proteobacteria	Gammaproteobacteria	Xanthomonadales
O14	Firmicutes	Clostridia	Clostridiales
O15	Acidobacteriota	Blastocatellia	DS-100
O16	Elusimicrobiota	Elusimicrobia	Lineage IV
O17	Bacteroidota	Bacteroidia	Chitinophagales
O18	Proteobacteria	Gammaproteobacteria	Unclassified
O19	Acidobacteriota	Acidobacteriae	PAUC26f
O20	Proteobacteria	Gammaproteobacteria	Burkholderiales
O21	Myxococcota	Polyangia	Unclassified
O22	Bacteroidota	Bacteroidia	Flavobacteriales
O23	Acidobacteriota	Acidobacteriae	Acidobacteriales
O24	GAL15	Unclassified	Unclassified
O25	Cyanobacteria	Vampirivibrionia	Obscuribacterales
O26	Unclassified	Unclassified	Unclassified
O27	Acidobacteriota	Acidobacteriae	Subgroup 2
O28	Proteobacteria	Alphaproteobacteria	Rhodobacterales
O29	Methylomirabilota	Methylomirabilia	Rokubacteriales
O30	Acidobacteriota	Acidobacteriae	Subgroup 13
O31	Actinobacteriota	Acidimicrobiia	IMCC26256
O32	Bacteroidota	Bacteroidia	Sphingobacteriales
O33	Chloroflexi	Anaerolineae	RBG-13-54-9
O34	Planctomycetota	Unclassified	Unclassified
O35	Chloroflexi	Ktedonobacteria	B12-WMSP1
O36	Proteobacteria	Gammaproteobacteria	Vibrionales
O37	Chloroflexi	Ktedonobacteria	Ktedonobacterales
O38	Planctomycetota	Pla4 lineage	Unclassified
O39	Actinobacteriota	Thermoleophilia	Solirubrobacterales
O40	Proteobacteria	Alphaproteobacteria	Unclassified
O41	Proteobacteria	Gammaproteobacteria	Gammaproteobacteria Incertae Sedis
O42	Actinobacteriota	Actinobacteria	Pseudonocardiales
O44	RCP2-54	Unclassified	Unclassified
O45	Proteobacteria	Alphaproteobacteria	Acetobacterales
O46	Proteobacteria	Gammaproteobacteria	Coxiellales
O47	Chloroflexi	Unclassified	Unclassified
O48	Patescibacteria	ABY1	Unclassified
O49	Myxococcota	Polyangia	Haliangiales
O51	Crenarchaeota	Nitrososphaeria	Nitrosotaleales
O52	Chloroflexi	Chloroflexia	Unclassified
O53	Firmicutes	Bacilli	Bacillales
O55	Proteobacteria	Gammaproteobacteria	WD260
O56	Chloroflexi	AD3	Unclassified
O57	Proteobacteria	Alphaproteobacteria	Sphingomonadales
O58	Proteobacteria	Gammaproteobacteria	Salinisphaerales
O59	Myxococcota	Polyangia	Polyangiales
O60	Actinobacteriota	Actinobacteria	Frankiales
O61	Proteobacteria	Alphaproteobacteria	Rickettsiales
O62	WPS-2	Unclassified	Unclassified
O63	Verrucomicrobiota	Verrucomicrobiae	Opitutales
O64	Gemmatimonadota	BD2-11 terrestrial group	Unclassified
O65	Planctomycetota	Planctomycetes	Isosphaerales
O66	Chloroflexi	Chloroflexia	Thermomicrobiales
O67	Acidobacteriota	Acidobacteriae	Unclassified
O68	Bacteroidota	Bacteroidia	Cytophagales
O69	Verrucomicrobiota	Chlamydiae	Chlamydiales
O70	Verrucomicrobiota	Verrucomicrobiae	Chthoniobacterales
O71	Proteobacteria	Alphaproteobacteria	Dongiales
O72	Proteobacteria	Gammaproteobacteria	Pseudomonadales
O73	Chloroflexi	Anaerolineae	SBR1031
O74	Planctomycetota	Planctomycetes	Pirellulales

O75	Planctomycetota	vadinHA49	Unclassified
O76	Proteobacteria	Unclassified	Unclassified
O77	Proteobacteria	Gammaproteobacteria	Legionellales
O78	Proteobacteria	Gammaproteobacteria	Enterobacterales
O79	Desulfobacterota	uncultured	Unclassified
O80	Chloroflexi	JG30-KF-CM66	Unclassified
O81	Gemmatimonadota	Gemmatimonadetes	Gemmatimonadales
O82	Acidobacteriota	Acidobacteriae	Solibacterales
O83	Proteobacteria	Alphaproteobacteria	Micropepsales
O84	Dependentiae	Babeliae	Babeliales
O85	Bacteroidota	Bacteroidia	Bacteroidales
O86	Crenarchaeota	Nitrososphaeria	Group 1.1c
O87	MBNT15	Unclassified	Unclassified
O88	Chloroflexi	OLB14	Unclassified
O89	Proteobacteria	Alphaproteobacteria	Caulobacterales
O90	Acidobacteriota	Holophagae	Subgroup 7
O91	Bdellovibrionota	Oligoflexia	0319-6G20
O92	Proteobacteria	Gammaproteobacteria	Cellvibrionales
O93	Planctomycetota	BD7-11	Unclassified
O94	Acidobacteriota	Blastocatellia	Unclassified
O95	Entotheonellaeota	Entotheonellia	Entotheonellales
O96	Cyanobacteria	Cyanobacteriia	Chloroplast
O97	Proteobacteria	Gammaproteobacteria	Methylococcales
O98	Nitrospirota	Nitrospiria	Nitrospirales
O99	Actinobacteriota	Acidimicrobiia	uncultured
O100	Patescibacteria	Saccharimonadia	Saccharimonadales
O101	Proteobacteria	Gammaproteobacteria	HOC36
O102	Thermoplasmatota	Thermoplasmata	uncultured
O103	Actinobacteriota	Actinobacteria	Corynebacteriales
O104	Thermotogota	Thermotogae	Kosmotogales
O105	Actinobacteriota	Actinobacteria	Propionibacteriales
O106	Chloroflexi	Anaerolineae	Anaerolineales
O107	Myxococcota	Myxococcia	Myxococcales
O108	Planctomycetota	Phycisphaerae	Unclassified
O109	Thermoplasmatota	Thermoplasmata	Methanomassiliicoccales
O110	Acidobacteriota	Subgroup 11	Unclassified
O111	Bacteroidota	Kapabacteria	Kapabacteriales
O112	Acidobacteriota	Thermoanaerobaculia	Thermoanaerobaculales
O113	Proteobacteria	Alphaproteobacteria	Holosporales
O114	Planctomycetota	Planctomycetes	Planctomycetales
O115	Firmicutes	Unclassified	Unclassified
O116	Firmicutes	Bacilli	Izemoplasmatales
O117	Planctomycetota	Phycisphaerae	Phycisphaerales
O118	Armatimonadota	Fimbriimonadia	Fimbriimonadales
O119	Proteobacteria	Gammaproteobacteria	Aeromonadales
O120	Armatimonadota	uncultured	Unclassified
O121	Actinobacteriota	Thermoleophilia	Gaiellales
O122	Proteobacteria	Gammaproteobacteria	Diplorickettsiales
O123	Chloroflexi	Anaerolineae	Ardenticatenales
O124	Verrucomicrobiota	Verrucomicrobiae	Pedosphaerales
O125	Proteobacteria	Alphaproteobacteria	Reyranellales
O126	Proteobacteria	Alphaproteobacteria	Paracaedibacterales
O127	Chloroflexi	Dehalococcoidia	S085
O128	Actinobacteriota	Actinobacteria	Streptomycetales
O129	Acidobacteriota	Unclassified	Unclassified
O130	Bacteroidota	Unclassified	Unclassified
O131	Planctomycetota	Phycisphaerae	Tepidisphaerales
O132	Actinobacteriota	Actinobacteria	Micrococcales
O133	Elusimicrobiota	Lineage IIa	Unclassified
O134	Bdellovibrionota	Bdellovibrionia	Bacteriovoracales
O135	Planctomycetota	OM190	Unclassified
O136	Acidobacteriota	Blastocatellia	Blastocatellales
O137	Sva0485	Unclassified	Unclassified
O138	Chloroflexi	Dehalococcoidia	SAR202 clade
O140	Firmicutes	Bacilli	Alicyclobacillales
O141	Verrucomicrobiota	Verrucomicrobiae	S-BQ2-57 soil group
O142	Acidobacteriota	Vicinamibacteria	Subgroup 17
O143	Proteobacteria	Gammaproteobacteria	Acidithiobacillales
O144	Bacteroidota	SJA-28	Unclassified
O145	Actinobacteriota	Actinobacteria	Catenulisporales
O146	Acidobacteriota	Acidobacteriae	Subgroup 12
O147	Thermoplasmatota	Thermoplasmata	Thermoplasmatales
O148	Armatimonadota	Chthonomonadetes	Chthonomonadales

O149	Chloroflexi	Anaerolineae	Caldilineales
O150	Proteobacteria	Gammaproteobacteria	Alteromonadales
O151	Firmicutes	Limnochordia	MBA03
O152	Proteobacteria	Gammaproteobacteria	JG36-GS-52
O153	Spirochaetota	Leptospirae	Leptospirales
O154	Firmicutes	Bacilli	Lactobacillales
O155	Chloroflexi	TK10	Unclassified
O156	Firmicutes	Bacilli	Erysipelotrichales
O157	Chloroflexi	P2-11E	Unclassified
O158	Firmicutes	Bacilli	Entomoplasmatales
O159	Firmicutes	Clostridia	Eubacteriales
O160	Firmicutes	Clostridia	Peptostreptococcales-Tissierellales
O161	Myxococcota	Polyangia	mle1-27
O162	Euryarchaeota	Methanobacteria	Methanobacteriales
O163	Firmicutes	Bacilli	Acholeplasmatales
O164	Armatimonadota	Armatimonadia	Armatimonadales
O165	Firmicutes	Incertae Sedis	DTU014
O166	Firmicutes	Clostridia	Peptococcales
O167	Verrucomicrobiota	Omnitrophia	Omnitrophales
O168	Myxococcota	bacteriap25	Unclassified
O169	Proteobacteria	Alphaproteobacteria	Azospirillales
O170	Firmicutes	Negativicutes	Veillonellales-Selenomonadales
O171	Dadabacteria	Dadabacteriia	Dadabacteriales
O172	Desulfobacterota	Desulfuromonadia	Geobacterales
O173	Elusimicrobiota	Lineage IIc	Unclassified
O174	Crenarchaeota	Bathyarchaeia	Unclassified
O175	Proteobacteria	Gammaproteobacteria	JG36-TzT-191
O176	Acidobacteriota	Subgroup 18	Unclassified
O177	Elusimicrobiota	Endomicrobia	Endomicrobiales
O178	Actinobacteriota	Actinobacteria	Streptosporangiales
O179	Nitrospirota	Leptospirillia	Leptospirillales
O180	Proteobacteria	Alphaproteobacteria	Kordiimonadales
O181	Firmicutes	Bacilli	Paenibacillales
O182	Planctomycetota	Phycisphaerae	mle1-8
O183	Desulfobacterota	Syntrophia	Syntrophales
O184	Acidobacteriota	Subgroup 5	Unclassified
O185	Cyanobacteria	Cyanobacteriia	Synechococcales
O186	Proteobacteria	Gammaproteobacteria	Halothiobacillales
O187	Cyanobacteria	Sericytochromatia	Unclassified
O188	Acidobacteriota	Blastocatellia	Pyrinomonadales
O189	Spirochaetota	Spirochaetia	Spirochaetales
O190	Proteobacteria	Alphaproteobacteria	Caedibacterales
O191	Proteobacteria	Alphaproteobacteria	Puniceispirillales
O192	Desulfobacterota	Unclassified	Unclassified
O193	Firmicutes	Clostridia	Oscillospirales
O194	Deinococcota	Deinococci	Thermales
O195	Firmicutes	Syntrophomonadia	Syntrophomonadales
O197	Acidobacteriota	Holophagae	Holophagales
O198	Proteobacteria	Gammaproteobacteria	Steroidobacterales
O199	Cyanobacteria	Vampirivibrionia	Vampirovibrionales
O200	Fusobacteriota	Fusobacteriia	Fusobacteriales
O201	Firmicutes	Clostridia	Gracilibacteraceae
O202	Proteobacteria	Gammaproteobacteria	Ga0077536
O203	Proteobacteria	Alphaproteobacteria	SAR11 clade
O204	Campilobacterota	Campylobacteria	Campylobacterales
O205	Proteobacteria	Gammaproteobacteria	KF-JG30-C25
O206	Planctomycetota	Phycisphaerae	S-70
O207	Bacteroidota	Rhodothermia	Balneolales
O208	Chloroflexi	Anaerolineae	Unclassified
O209	Firmicutes	Bacilli	RF39
O210	Elusimicrobiota	Elusimicrobia	Unclassified
O211	Synergistota	Synergistia	Synergistales
O212	Myxococcota	Polyangia	Nannocystales
O213	Methylomirabilota	Methylomirabilia	Methylomirabilales
O214	Firmicutes	Clostridia	Christensenellales
O215	Myxococcota	Unclassified	Unclassified
O216	Firmicutes	Thermincolia	Carboxydocellales
O217	Chloroflexi	KD4-96	Unclassified
O219	Acidobacteriota	Acidobacteriae	Subgroup 15
O220	SAR324 clade(Marine group B)	Unclassified	Unclassified
O221	Bacteroidota	Bacteroidia	Unclassified
O222	Crenarchaeota	Nitrososphaeria	Nitrososphaerales
O223	Cloacimonadota	Cloacimonadia	Cloacimonadales
O224	Patescibacteria	Parcubacteria	Unclassified
O225	Verrucomicrobiota	Verrucomicrobiae	Methylacidiphilales
O226	Desulfobacterota	Desulfobulbia	Desulfobulbales

O227	Desulfobacterota	Desulfovibrionia	Desulfovibrionales
O228	Cyanobacteria	Cyanobacteriia	Cyanobacteriales
O229	Desulfobacterota	Syntrophobacteria	Syntrophobacterales
O230	Patescibacteria	Gracilibacteria	Unclassified
O231	Proteobacteria	Gammaproteobacteria	uncultured
O232	Calditrichota	Calditrichia	Calditrichales
O233	Actinobacteriota	Acidimicrobiia	Microtrichales
O234	Nanoarchaeota	Nanoarchaeia	Woesearchaeales
O235	Proteobacteria	Gammaproteobacteria	Nitrosococcales
O236	Firmicutes	Bacilli	Staphylococcales
O237	Actinobacteriota	Unclassified	Unclassified
O238	Elusimicrobiota	Unclassified	Unclassified
O240	Firmicutes	Bacilli	Exiguobacterales
O241	Crenarchaeota	Nitrososphaeria	uncultured
O242	Patescibacteria	Microgenomatia	Candidatus Woykebacteria
O244	Bacteroidota	Kryptonia	Kryptoniales
O245	Firmicutes	Clostridia	Lachnospirales
O246	Firmicutes	Clostridia	Unclassified
O247	Verrucomicrobiota	Verrucomicrobiae	Unclassified
O248	Latescibacterota	Unclassified	Unclassified
O249	Proteobacteria	Alphaproteobacteria	Thalassobaculales
O250	Planctomycetota	Planctomycetes	Unclassified
O251	Chloroflexi	SHA-26	Unclassified
O252	Elusimicrobiota	Lineage IIb	Unclassified
O253	Acidobacteriota	Blastocatellia	Unclassified
O254	Proteobacteria	Gammaproteobacteria	CCD24
O255	Patescibacteria	Dojkabacteria	Unclassified
O256	Planctomycetota	Phycisphaerae	CCM11a
O257	Firmicutes	Negativicutes	Acidaminococcales
O258	Proteobacteria	Alphaproteobacteria	Micavibrionales
O259	Abditibacteriota	Abditibacteria	Abditibacteriales
O261	Cyanobacteria	Cyanobacteriia	Leptolyngbyales
O262	Patescibacteria	Microgenomatia	Candidatus Woesebacteria
O263	Acidobacteriota	Acidobacteriae	Paludibaculum
O264	Proteobacteria	Gammaproteobacteria	Oceanospirillales
O265	Proteobacteria	Alphaproteobacteria	Tistrellales
O266	Cyanobacteria	Cyanobacteriia	Phormidesmiales
O267	Firmicutes	Bacilli	Unclassified
O268	Patescibacteria	Parcubacteria	Candidatus Adlerbacteria
O269	Myxococcota	Polyangia	Blfdi19
O270	Actinobacteriota	MB-A2-108	Unclassified
O271	Proteobacteria	Alphaproteobacteria	Defluviicoccales
O272	Gemmatimonadota	PAUC43f marine benthic group	Unclassified
O273	Thermoplasmatota	Thermoplasmata	Marine Group II
O274	Sumerlaeota	Sumerlaeia	Sumerlaeales
O275	Micrarchaeota	Micrarchaeia	Micrarchaeales
O276	Proteobacteria	Gammaproteobacteria	Immundisolibacterales
O277	Nitrospirota	Thermodesulfovibrionia	uncultured

Supplementary Table S4. Most abundant ASVs affiliated to the significantly enriched bacterial phyla of DRY, WET, and WATER groups and their Best BLAST Hits from the GenBank database[$,#]

WET					
**ASV**	**Taxon***	**Best BLAST Hit**	**Acc Num**	**Isolation source**	**Id %**
64	o_Subgroup 2	Uncultured bacterium clone BS 7640D03	KU884066	soil from sugarcane	100
684	o_Subgroup 13	Uncultured forest soil bacterium clone DUNssu389	AY913561	forest soil	99.55
806	o_Subgroup 2	16S rRNA amplicon fragment from a soil sample (ferralsol, Madagascar)	FQ747929	ferralsol	100
854	o_Subgroup 2	Uncultured bacterium clone FL4Bd9_7464	JQ373565	FACE soil	100
891	o_Subgroup 13	Uncultured bacterium clone HD60	JX885278	forest soil	99.11
1090	o_Subgroup 2	Uncultured bacterium clone OTU268	KT785055	rhizosphere soil	100
1120	o_Subgroup 13	Uncultured bacterium clone OTU_32	MK724088	soil	100
1978	o_Subgroup 13	Uncultured bacterium clone OTU_32	MK724088	soil	99.55
2096	o_Acidobacteriales	Uncultured bacterium clone B074	JX967645	alpine soil	100
2277	o_Subgroup 2	Uncultured bacterium clone WLCLC436	JN168405	forest soil	99.55
2334	g_Acidipila	Uncultured bacterium clone NCD1s1Powerc5_4910	JQ382441	FACE soil	99.55
2470	o_Acidobacteriales	Uncultured bacterium clone GB92742hO9	JN643106	lava tube wall	100
2558	o_Subgroup 2	Uncultured bacterium clone OTU_4612	MH528260	bamboo forest soil	99.11
2683	o_Subgroup 2	Uncultured bacterium clone H10_2226571	LT720601	marine water	99.55
3589	o_Subgroup 2	Uncultured soil bacterium clone 21_77KF11	GQ918990	subtropical Altamaha and Ohopee River Valley eolian chronosequences ages 4,910 to 77,400 years	97.77
3778	g_Granulicella	Uncultured bacterium clone NCD3s1Powerb5_5580	JQ382095	FACE soil	100
3862	o_Subgroup 2	16S rRNA amplicon fragment from a soil sample (ferralsol, Madagascar)	FQ680713	ferralsol	99.55
4034	f_Acidobacteriaceae	Edaphobacter acidisoli strain 4G-K17	NR_158119	forest soil	100
4230	o_Subgroup 2	Uncultured bacterium clone TSC87	EU359997	Tatachia forest	100

4371	o_Subgroup 2	Uncultured bacterium clone SLSTR371	JN851520	Shan Lin Si forest	100
4453	o_Subgroup 7	Uncultured bacterium clone OTU_551	MH524199	forest soil	100
4497	o_Acidobacteriales	Uncultured bacterium clone E187	JF833892	potassium mine soil	96.88
4694	o_Subgroup 13	Uncultured bacterium clone HDB_SIPI490	HM187010	saturated zone of the Hanford Site 300 Area subsurface	98.66
4849	o_Subgroup 2	Uncultured bacterium clone GZ081025	KJ740470	paddy soil	100
5043	o_Acidobacteriales	Uncultured bacterium clone NTDAS10366	KJ407909	forest soil	98.66
5059	o_Acidobacteriales	Uncultured Acidobacteria bacterium clone SEW_08_122	HQ598848	woodland soil	100
5457	o_Subgroup 2	Uncultured bacterium clone BO654O2dO4	JN850181	white microbial mat from lava tube wall	100
5685	o_Acidobacteriales	Uncultured bacterium clone NTDAS8387	KJ407684	forest soil	99.55
5746	o_Subgroup 2	Uncultured bacterium clone C3 E47	GU366801	temperate forest soil	98.21
**DRY**					
19	c_Acidimicrobiia	Uncultured Acidimicrobiales bacterium clone KUI-17-28	MT604950	sediment of acidic hot spring	96.43
203	g_Acidothermus	Uncultured bacterium clone L3_W8_B4_C3	KT819102	forest soil	100
304	g_Mycobacterium	Uncultured bacterium clone Qb_109f_73	MK527799	cerrado soil (acidic and poor nutrient soil)	100
322	g_Mycobacterium	Uncultured bacterium clone Qb_109f_73	MK527799	cerrado soil (acidic and poor nutrient soil)	99.55
473	g_Conexibacter	16S rRNA amplicon fragment from a soil sample (ferralsol, Madagascar)	FQ763736	ferralsol	96.88
562	g_Mycobacterium	Uncultured bacterium clone Qb_109f_73	MK527799	cerrado soil (acidic and poor nutrient soil)	99.11
799	c_Actinobacteria	16S rRNA amplicon fragment from a soil sample (ferralsol, Madagascar)	FQ684229	ferralsol	96.85
1222	g_Crossiella	Uncultured bacterium clone FL4Bh5_11724	JQ377933	FACE soil	98.21
1292	g_Crossiella	Uncultured bacterium clone FL4Bh5_11724	JQ377933	FACE soil	97.77
1356	g_Acidothermus	16S rRNA amplicon fragment from a soil sample (ferralsol, Madagascar)	FQ745392	ferralsol	95.54

1639	o_IMCC26256	Uncultured bacterium clone FL4Bd9_10695	JQ376488	FACE soil	97.32
1990	g_Crossiella	Uncultured actinobacterium clone RSC_RRA02	GU205301	Roraima Sur Cave	99.55
2061	c_Acidimicrobiia	Uncultured Acidimicrobiales bacterium clone KUI-17-28	MT604950	sediment of acidic hot spring	96.88
2392	g_Acidothermus	16S rRNA amplicon fragment from a soil sample (ferralsol, Madagascar)	FQ745392	ferralsol	96.43
2597	g_Mycobacterium	Uncultured bacterium clone Qb_109f_73	MK527799	cerrado soil (acidic and poor nutrient soil)	99.55
2613	g_Crossiella	Uncultured bacterium clone FL4Bh5_11724	JQ377933	FACE soil	99.11
2704	c_Acidimicrobiia	Uncultured bacterium clone FL4Bd9_10695	JQ376488	FACE soil	96.88
2713	c_Acidimicrobiia	Uncultured bacterium clone W2bXIb49	EU419187	acidic, hydrothermally modified volcanic soil	97.32
2997	g_Acidothermus	16S rRNA amplicon fragment from a soil sample (ferralsol, Madagascar)	FQ745392	ferralsol	97.32
3625	g_Conexibacter	Uncultured bacterium clone PB17007-1_G01	JX171801	Antarctic soil, glacier forefield	99.55
3763	g_Acidothermus	16S rRNA amplicon fragment from a soil sample (ferralsol, Madagascar)	FQ745392	ferralsol	95.54
3838	c_Acidimicrobiia	16S rRNA amplicon fragment from a soil sample (ferralsol, Madagascar)	FQ713685	ferralsol	97.32
3893	g_Crossiella	Uncultured actinobacterium clone RSC_RRA02	GU205301	Roraima Sur Cave	98.21
4081	g_Acidothermus	16S rRNA amplicon fragment from a soil sample (ferralsol, Madagascar)	FQ745392	ferralsol	95.09
4297	o_IMCC26256	Uncultured Acidimicrobiales bacterium clone KUI-17-28	MT604950	sediment of acidic hot spring	96.88
4386	g_Acidothermus	16S rRNA amplicon fragment from a soil sample (ferralsol, Madagascar)	FQ745392	ferralsol	96.88
4524	c_Actinobacteria	16S rRNA amplicon fragment from a soil sample (ferralsol, Madagascar)	FQ684229	ferralsol	97.3
4885	g_Mycobacterium	Uncultured bacterium clone BOTU631	MG580500	Mariana trench	99.11
5516	o_IMCC26256	Uncultured bacterium clone FL4Bd9_10695	JQ376488	FACE soil	97.32
**WATER**					
172	o_Burkholderiales	Uncultured beta proteobacterium clone 1505	KX367409	Lake Taihu	100
513	f_Yersiniaceae	Serratia myotis strain CsMH-309	MT415189	cave stalactite	100

879	f_Enterobacteriaceae	Enterobacter roggenkampii strain RHBSTW-00002	CP058196	wastewater influent sample	100
1118	g_Pseudomonas	Pseudomonas extremaustralis strain WS-1	MT641229	swampy soil of Western Siberia	100
1263	g_Delftia	Delftia sp. strain W9-4-1	MG561180	Dianchi Lake	100
1616	g_NMD1	Uncultured bacterium clone FCPT489	EF516619	grassland soil	100
1934	g_Pseudomonas	Unidentified bacterium isolate La914j	LS483030	apse of Lascaux Cave	100
2165	c_Gammaproteobacteria	Uncultured bacterium clone OTU_1177	MH524825	forest soil	100
2883	g_Stenotrophomonas	Stenotrophomonas pavanii strain S2-6	MT645777	Pulau Lang Tengah in Malaysia	99.55
3619	g_Serratia	Serratia marcescens strain N1.14	MT645673	surface-sterilized roots of the Cd-hyperaccumulator Solanum nigrum	100
3648	f_Nitrosomonadaceae	Uncultured bacterium clone TE2c1530d6 8457	JQ374372	FACE soil	100
3772	g_Pantoea	Pantoea agglomerans strain CLS10	ON359893	rhizosphere	100
4040	g_Acinetobacter	Acinetobacter seifertii strain WS1	MT632639	swampy soil of Western Siberia	100
4662	f_Oxalobacteraceae	Janthinobacterium sp. strain RRC54	MK965188	Raspberry Rising Cave	100
5348	g_Stenotrophomonas	Stenotrophomonas maltophilia strain SH4	MT256163	water from the ghadikola lagoon	100
5640	f_Enterobacteriaceae	Enterobacteriaceae bacterium Ri2Pw 5895	AB833101	Toyohira river water	100
5667	g_Stenotrophomonas	Stenotrophomonas sp. SKD-GYT-1	LC479453	heavy metal contaminated soil	100

[$] The top 5 most abundant ASVs for each sample (in each group) have been included in the analysis
[#] The Best BLAST Hit of each ASV retrieved from GenBank database through BLASTn analysis is provided as well as the Accession number and the isolation source
[*] The lowest taxonomy level retrieved by Silva for each dominant ASV

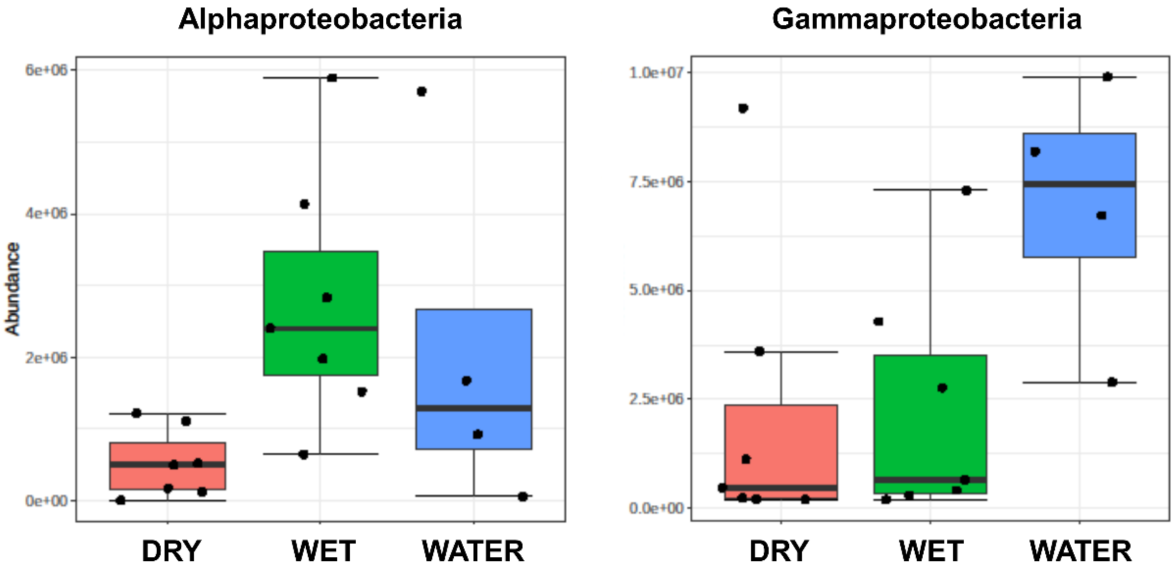

Supplementary Figure S1. Rank test of *Alphaproteobacteria* and *Gammaproteobacteria* classes.

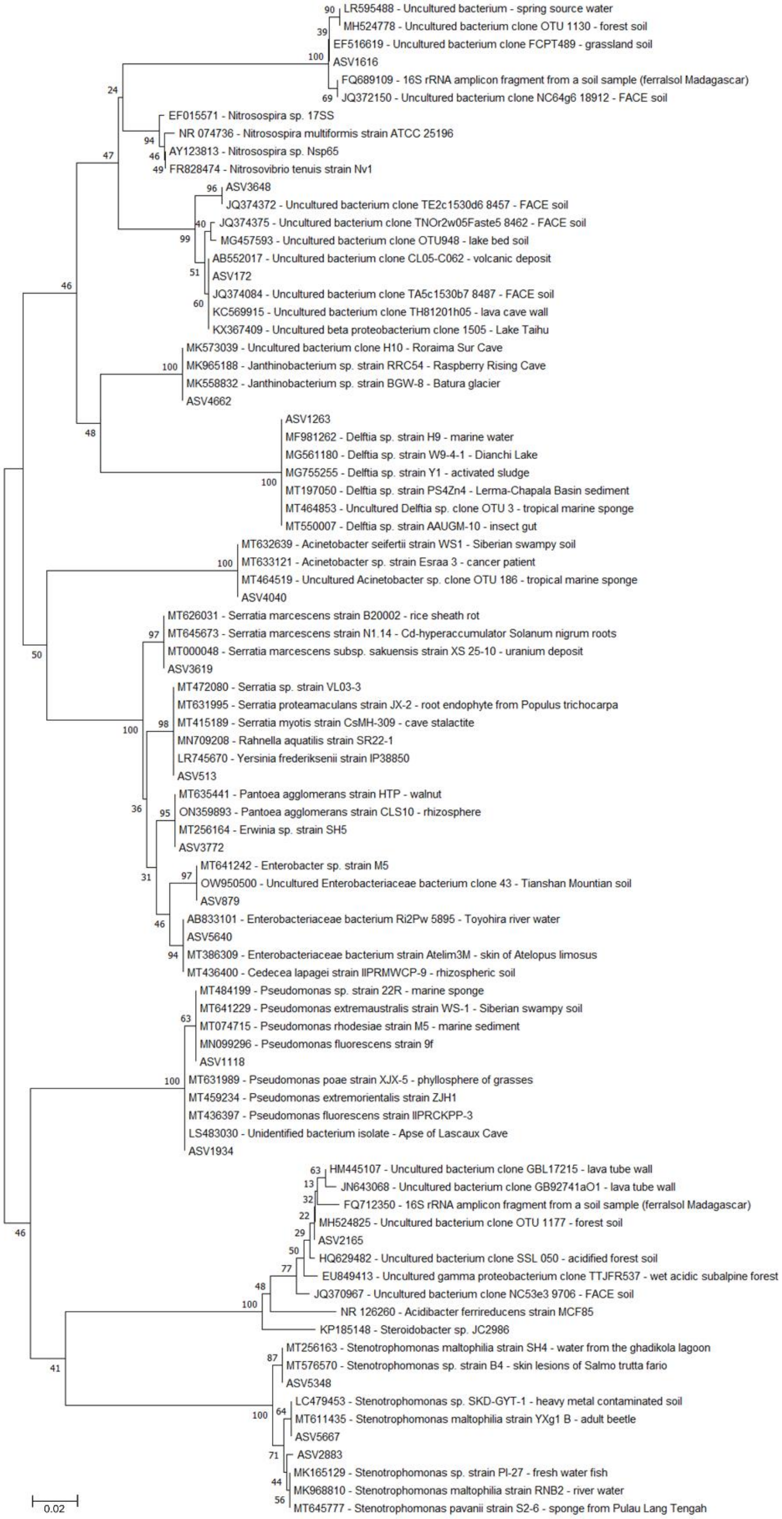

Supplementary Figure S2. Neighbor-joining phylogenetic tree including the most abundant ASVs affiliated to the *Gammaproteobacteria* class in WATER samples along with the Best BLAST Hits retrieved from the GenBank database. The numbers on the nodes indicate bootstrap values.

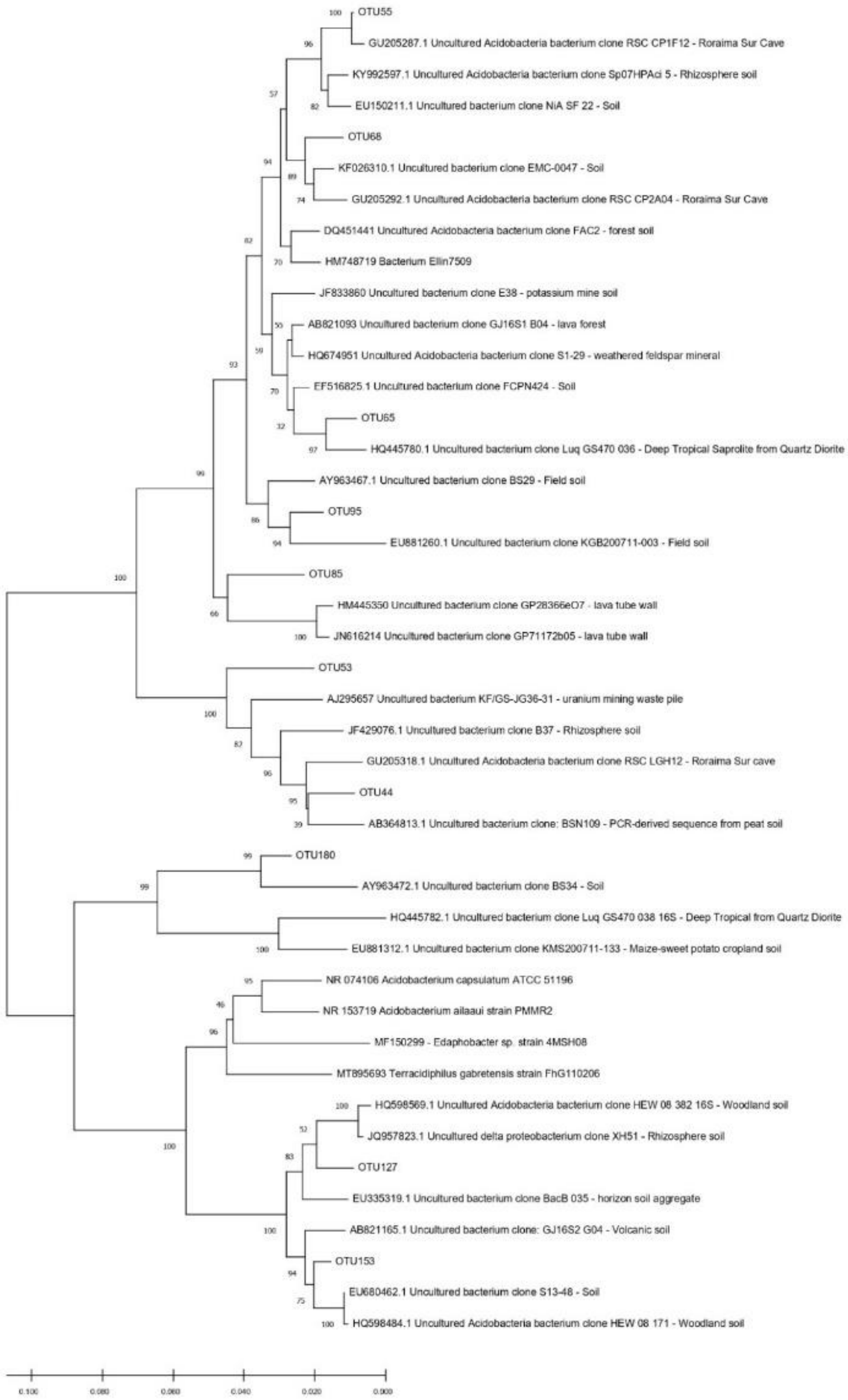

Supplementary Figure S3. Neighbor-joining phylogenetic tree including the most abundant EMIRGE-derived OTUs affiliated to the *Acidobacteriota* phylum of the WET samples from Imawarì Yeuta cave along with the Best BLAST Hits retrieved from the GenBank database. The numbers on the nodes indicate bootstrap values.

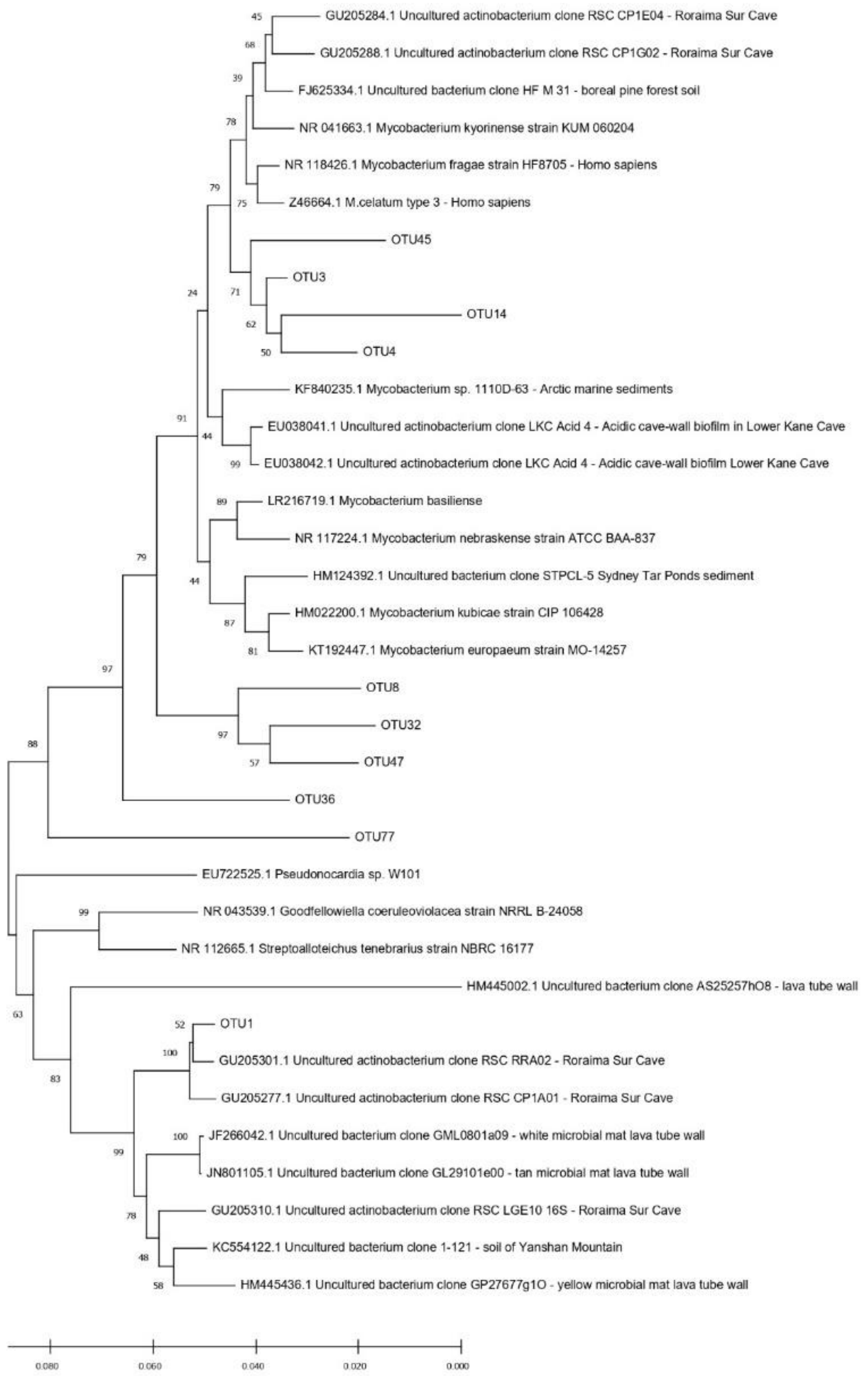

Supplementary Figure S4. Neighbor-joining phylogenetic tree including the most abundant EMIRGE-derived OTUs affiliated to the *Actinobacteriota* phylum from DRY samples of Imawarì Yeuta along with the Best BLAST Hits retrieved from the GenBank database. The numbers on the nodes indicate bootstrap values.
